# Will Robots Automate Your Job Away? Full Employment, Basic Income and Economic Democracy

**DOI:** 10.1093/indlaw/dwab010

**Published:** 2021-07-14

**Authors:** Ewan McGaughey

**Affiliations:** 1 King’s College, London; 2 Centre for Business Research, University of Cambridge

## Abstract

Will the internet, robotics and artificial intelligence mean a ‘jobless future’? A recent narrative, endorsed by tech-billionaires, says we face mass unemployment, and we need a basic income. In contrast, this article shows why the law can achieve full employment with fair incomes, and holidays with pay. Universal human rights, including the right to ‘share in scientific advancement and its benefits’, set the proper guiding principles. Three distinct views of the causes of unemployment are that it is a ‘natural’ phenomenon, that technology may propel it, or that it is social and legal choice: to let capital owners restrict investment in jobs. Only the third view has any credible evidence to support it. Technology may create redundancies, but unemployment is a purely social phenomenon. After World War Two, 42% of UK jobs were redundant but social policy maintained full employment. This said, transition to new technology, when markets are left alone, can be exceedingly slow: a staggering 88% of American horses lost their jobs after the Model T Ford, but only over 45 years. Both the global financial crisis from 2008 and the COVID-19 pandemic from 2020 illustrate the importance of social and legal policy, and suggest it is time to learn. Taking lessons from history, it is clear that unemployment is driven by inequality of wealth and of votes in the economy. To uphold human rights, governments should reprogramme the law, for full employment, fair incomes and more leisure, on a living planet. Robot owners will not automate your job away, if we defend economic democracy.

## 1. INTRODUCTION

Today’s technology enables us to make a paradise on Earth, where scarcity and poverty will be forgotten, and yet we remain consumed by visions of dystopia. As inequality has become more extreme, a busload of billionaires own more wealth than half the planet,^1^ and many of them are saying that new technology (that they aim to own) will mean mass unemployment. The solution they propose is a basic income. Facebook owner, Mark Zuckerberg, said that ‘technology and automation are eliminating many jobs’ and we ‘should explore ideas like universal basic income to give everyone a cushion to try new things’.^2^ Tesla owner, Elon Musk, said, ‘Twenty years is a short period of time to have something like 12–15 percent of the workforce be unemployed’ and a basic income is ‘going to be necessary’ because there ‘will be fewer and fewer jobs that a robot cannot do better’.^3^ The foundation of eBay’s Pierre Omidyar, says ‘automation is replacing traditional jobs’, and the gig economy ‘may make employment far less stable and reliable for supporting a livelihood’.^4^ Advocates of this narrative respond sharply to criticism: a contributor to the Washington Post, owned by Amazon billionaire Jeff Bezos, wrote ‘Sorry, but the jobless future isn’t a luddite fallacy’.^5^

This narrative runs contrary to the often forgotten universal human right to ‘share in scientific advancement and its benefits’,^6^ but there is intellectual support for it in economics and sociology.^7^ A basic income (if not its tax source) enjoys cross-ideological support from Milton Friedman to Guy Standing.^8^ A basic income can be seen as a way to decrease welfare cost, or social stigma. Some think it could eliminate the collective welfare state, others that it completes the welfare state. But once the shape of a basic income plan is agreed, we still must ask, is ‘automation’ the right reason for it? In 2013, two researchers named Cark Benedikt Frey and Michael Osborne dramatically stated that ‘47 per cent of total US employment’ is ‘potentially’ at risk from automation ‘over some unspecified number of years’.^9^ From 2015 they developed this argument with Citibank Research.^10^ Another paper in 2017 (part-funded by a Facebook billionaire) claimed that robots will replace most human functions and write a bestselling book by 2049. As with Frey and Osborne, its method included a survey, in this case of 352 ‘AI experts’.^11^ These figures (if not the reasoning) reached viral proportions in social media, and academia, with citations mounting as if ‘likes’ or ‘retweets’. The problem is that predictions of the future, ‘over some unspecified number of years’, are unfalsifiable. Like saying there is an ‘end of history’,^12^ or ‘skateboards might fly’,^13^ this ‘technological forecasting’ has all the shiny appeal, but also the predictive integrity, of a crystal ball. Tech may create redundancies, but unemployment differs completely.

This article explores how legal policy can create full employment and fair incomes, not just mass unemployment and basic incomes. Part 2 discusses three main views of the causes of unemployment. One says unemployment is ‘natural’, and full employment will accelerate inflation. There was never any credible evidence to support this view. A second view says that technology can and does drive unemployment. But it has become acutely clear that, even leaving aside the profound methodological flaws behind this theory, both the global financial crisis and the COVID-19 pandemic illustrate that social and legal policy can handle mass shocks, whether based on tech or anything else. A third view, less discussed outside times of crisis,^14^ says unemployment is a choice. Law can prevent or enable restrictions on the supply of capital to the job market and guarantee full employment. This view matches the Universal Declaration of Human Rights, with the right of everyone to work, to fair wages, leisure time, and social security, but also to ‘share in scientific advancement and its benefits’.^15^ In its simplest dimensions, as the owners of *Animal Farm* soon knew,^16^ technology will mean social prosperity, if the gains are not trottered away by a few.

We cannot know the future, but we can learn from the past. Part 3 explores five periods of history where technology affected social relations, and the lessons that can be drawn. First, enclosures for sheep (maybe the first disruptive ‘app’) and the textile industry drove Luddite and Swing riots in Britain. This showed that when people are excluded from technology’s benefits, paid for by their labour, it causes unrest. Second, after WW2, demobilisation in the UK meant 42% of all workers, from armies to munitions factories, were redundant and had to move to civil production. The change was rapid, vast, and far more complex than automation. After post-WW1 chaos careful thought about *Full Employment in a Free Society* ensured that even such massive shocks were contained.^17^

Third, mass produced motors did mean mass redundancies: of horses. In the USA, a staggering 88% of horses lost their jobs, but only over 45 years. While driverless vehicles have rightly become a symbol of concern surrounding automation, human beings will not share the fate of horses, so long as humans, unlike horses, write laws. Yet this shows even if technological change is possible and profitable, free markets can be very slow. Fourth, the humble washing machine was a revolutionary technology, saving labour, improving gender equality. If people internalise the gains of automation, human development advances rapidly. Fifth, while artificial intelligence is remarkable, the evidence suggests computers will not develop cognitive functions.^18^ While the basis of a computer’s ‘intelligence’ is ‘0’s and ‘1’s, Cartesian self-awareness is far away. As teachers know, rote-learning is not critical thinking. Data is not knowledge. Doing is not understanding. Until science fiction becomes fact, and AI hype moves beyond clickbait, technology’s value will remain what it always has been: a way both to empower and accentuate the uniqueness of the human mind.

Unemployment is a purely social and legal phenomenon, yet our history and the evidence makes it clear that technological change can mean redundancies. So part 4 asks, does the law enable everyone to share fairly in technology’s gains? The answer is ‘not yet’. We can and should ‘reprogramme’ the law to uphold universal human rights, including the rights to work, to fair wages and to social security. First, the benefits of science and technology can be shared, with ‘just and favourable remuneration’ for everyone, by democratising the economy: guaranteeing the right to vote at work, in diversified capital funds (not concentrated share schemes), and in public services. Second, the law can guarantee universal social security for people who do not have traditional employment. A ‘universal basic income’ is a powerful idea that could fill gaps in the social security system, but it must not, like the disastrous Speenhamland system in the 19th century, subsidise poverty-pay employers. Third, the law can guarantee the right to work in cities and all regions and at the same time expand paid holidays by making corporate owners (not robots) pay their fair share of tax. These rights work together: they are indivisible. Part 5 concludes that robots will not automate your job away, if we defend economic democracy.

## 2. THREE VIEWS ON UNEMPLOYMENT’S CAUSES

Before discussing technology’s impact on jobs and how to respond to automation, it makes sense to engage the debate on unemployment’s causes in the 20th century. Political discourse often does the subject little credit. In one corner, it is often said the unemployed are responsible for unemployment, because they are lazy.^19^ In another, it is said there is a conscious plan to manufacture a reserve army of the unemployed, to press wages down.^20^ Credible theories tend to focus on objective factors: how social institutions and behaviour affect employment. They also acknowledge the desirability of full employment, and social security, as in the Universal Declaration of Human Rights of 1948.^21^ Three main theories are that (1) unemployment has a ‘natural’ rate, and attempts to go below accelerate inflation, (2) unemployment results from irrational human behaviour or maybe technology and (3) law determines employment by altering the supply of capital to the job market. Only the third view is backed by credible evidence.

### A. A ‘Natural’ Rate of Unemployment

A first main view, dominant in the late 20th century, is that unemployment has a ‘natural’ rate, worsened by labour or social rights. In 1967, as President of the American Economic Association, Milton Friedman argued the ‘natural’ rate was something that no government’s monetary or fiscal policy could sustainably reduce. Unemployment ‘can be kept below the “natural” rate only by accelerating inflation’.^22^ If monetary authorities tried to reduce unemployment below ‘3 per cent as the target rate’, but ‘the “natural” rate is higher than 3 per cent they will trigger inflation’.^23^ We ‘cannot know what the “natural” rate is’,^24^ said Friedman. But ‘legal minimum wage rates’, pro-labour public procurement and ‘the strength of labour unions’ made ‘the natural rate of unemployment higher than it would otherwise be’.^25^ Friedman argued that his theory followed from A.W. Phillips’ work in 1958. Phillips showed between 1861 and 1957, UK wages tended to rise when unemployment was low, and wages fell when unemployment was high.^26^ This confirmed an old logic, starting with Adam Smith that fuller employment raises worker bargaining power.^27^ If bargaining power is more equal, wages increase. Higher unemployment reduces worker power. But without any evidence, Friedman argued *all* inflation (not just wage rises) accelerates with full employment. For Friedman, this justified using monetary policy only to concentrate on price stability, apparently unconnected to full employment, and to undo all labour rights.^28^

Friedman’s theory was not new.^29^ In 1950, Friedrich von Hayek had already argued that if monetary policy or fiscal stimulus were used to boost enough demand to pay for ‘the kind of services…the unemployed offer’, it would have to be ‘of such a magnitude as to produce major inflationary effects’.^30^ There was no trade-off between unemployment and inflation, because if unemployment were suppressed below the natural rate, the acceleration of inflation would be out of control. Governments would then be driven, argued Hayek, ‘to control ever increasing parts of the economy’. Hayek did not provide evidence for this phenomenon because, with his bestselling *Road to Serfdom* in mind,^31^ the truth of his tale was ‘by now too well known to need elaboration’.^32^ This was the start of trying to find a supposed natural rate of unemployment which would not ‘accelerate’ inflation. The unknowable ‘natural’ unemployment rate became ‘NAIRU’—the ‘Non-Accelerating Inflation Rate of Unemployment’.^33^

**Figure 1.**
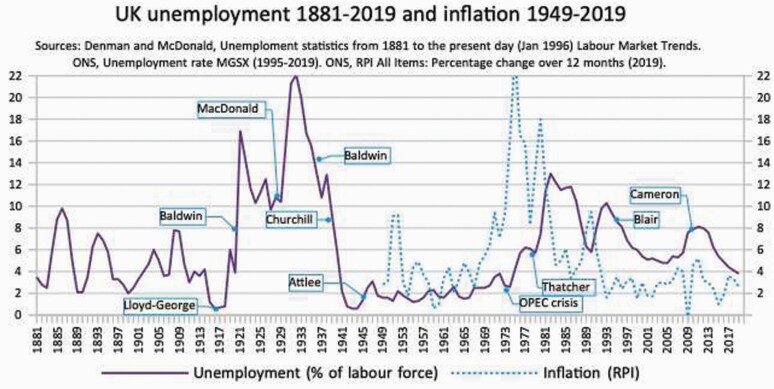
UK unemployment 1881–2019 and inflation 1949–2019. *Sources*: Denman and McDonald, ‘unemployment Statistics from 1881 to the present day (January 1996) Labour Market Trends’. ONS, Unemployment rate MGSX (1995–2019). ONS, ‘RPI All Times: Percentage change over 12 months (2019).

Among their followers, Friedman and Hayek seemed vindicated when the Organization of the Petroleum Exporting Countries unilaterally raised oil prices in 1973. The immediate trigger of the OPEC crisis was the Yom Kippur War, in the long and desperately sad Arab–Israeli conflict. Arab countries had attacked Israel in retaliation for Israel’s Six Day War in 1967. The US government sided with Israel. Arab oil producers raised their prices. As oil cost more, oil-dependent economies had less money to invest. As the costs were distributed through the market, there was stagnation, and few ways to avoid inflation: ‘stagflation’. Government practice had been to offset volatility in private and international investment with more spending. But now this policy had a price tag nobody could avoid. Despite being inflicted by despots,^34^ stagflation said to NAIRU disciples that full employment in a free society was impossible, without accelerating inflation. Certainly it was true that unemployment rose after OPEC. From 1945 to 1971, the UK had between 1.2 and 2.7%. Inflation from 1952 to 1967 averaged 3.12%.^35^ After OPEC, inflation surpassed 24%, and bad became the norm (see Figure 1).

But why when employment began to recover in 1977, did it stay so high in the 1980s and 90s? Unemployment in the UK reached 12.9% in 1983, and by 2020 barely went lower than 4%. The days of 1 to 2%, and full employment on fair wages without under-employment, seemed to be lost.

In 1977, Friedman developed his argument at a Bank of Sweden prize lecture, without evidence. Why were unemployment and inflation rising together? He said ‘there is no stable trade-off between inflation and unemployment’, but a natural rate of unemployment existed, consistent with real prices and ‘perceptions’.^36^ The natural rate had ‘clearly been rising’ because ‘women, teenagers and part-time workers’ were working, but moved jobs more, and unemployment insurance was ‘more generous’.^37^ He said before the OPEC crisis ‘in 1973, most countries show a clearly marked association of rising inflation and rising unemployment’, even though Friedman’s own charts showed that inflation was falling from 1970 to 1973 in five of seven countries.^38^ Nevertheless, for Friedman if inflation and unemployment now rose together (not an inverse, but a positive correlation) only a ‘modest elaboration of the natural-rate hypothesis’ was needed. This was ‘a temporary phenomenon that will disappear as economic agents adjust their expectations to reality’, though ‘we do not know what a complete adjustment will consist of’.^39^

**Figure 2.**
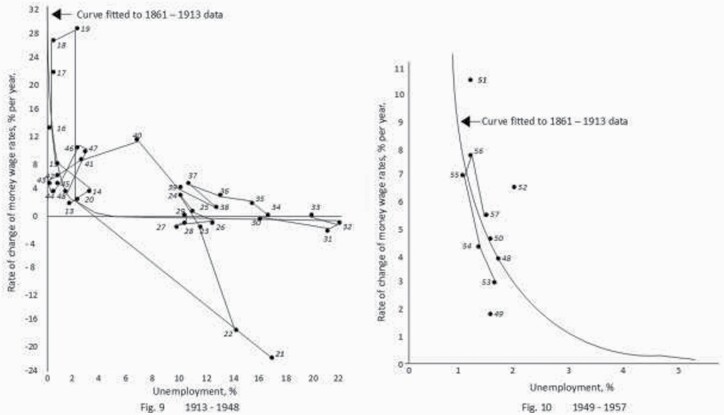
The curve of unemployment and wage rates from A. W. Phillips’ original charts.

**Figure 3.**
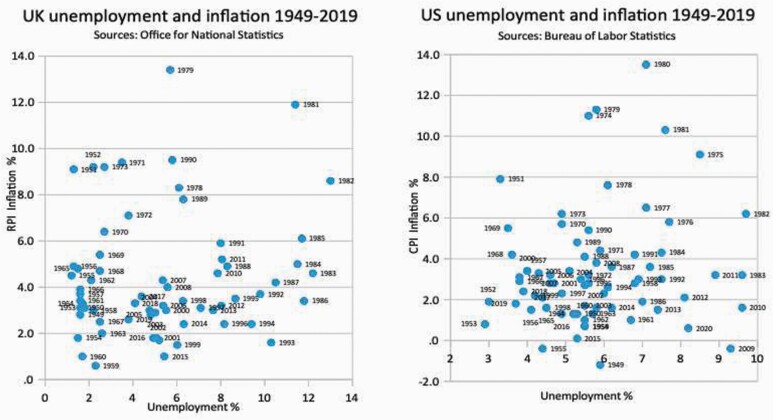
The random non-correlation of UK and US unemployment and inflation.

The ‘reality’ was that Friedman and Hayek’s theory was always evidence-free: a theory that held unless it did not. Clear in 1977,^40^ and clear now, the charts below show the logical correlation between fuller employment and higher wages in the Phillips curves (Figure 2). But despite the assertions of NAIRU theorists that ‘wage inflation is a crucial component of price inflation’,^41^ correlation between fuller employment and inflation never existed in the UK, USA, or anywhere (Figure 3). Full employment, rising wages and low inflation co-exist when production expands. Prices do not rise if people produce more: everyone’s welfare improves.

Many economists did oppose natural rate theory. In 1980, Shaun Hargreaves Heap explained that on its own logic the greater danger was to overshoot the (unknowable) natural unemployment rate: this would entrench higher unemployment, because people who are out of a job for a long time find it increasingly difficult to get back into work.^42^ Like a magnetic field lingers after the charge is switched off, this became known as ‘hysteresis’.^43^ In 1982, Kim Clark and Lawrence Summers, also rejecting a natural rate’s existence, explained how during WW2 in the USA, an unprecedented number of women entered the workforce whilst full employment was maintained. Unlike Friedman’s scapegoating of women or young people, Clark and Summers argued there is a tendency to find entering work harder after being out of work, and this contributes to more unemployment.^44^

But better theories did not stop NAIRU economics,^45^ any more than lack of evidence. In a special issue of the *Review of Economic Studies*, introduced by Richard Layard,^46^ it was said that natural unemployment was rising because ‘people are trying to achieve too high real wages’.^47^ The ‘rise in secular unemployment in Britain’, said Martyn Andrews and Stephen Nickell, was caused by benefits, moving workers, ‘the introduction of employment protection legislation and the rise in union power’.^48^ They asserted that the oil shocks caused but one third of 1973–77 inflation,^49^ again without evidence. By 1990, Samuel Bentolila and Giuseppe Bertola composed a table of employment protection laws in four countries (wholly unreferenced^50^) and were confident that these ‘costs’ were the cause of ‘Eurosclerosis’.^51^ Meanwhile, the Organization for Economic Cooperation and Development had begun regular reports to encourage cuts to job security.^52^ In 1994, Paul Krugman wrote that it was ‘conventional wisdom’ that the ‘disincentive effects of welfare state policies’ and the lack of ‘flexibility of the labour market’ causes unemployment.^53^ Eliminating social and job security might be ‘harsh’ and ‘some people end up on the scrap-heap’, but the ‘wisdom’ was clear: ‘Any tax or transfer payment distorts incentives’.^54^ In just 30 years, a generation of economists had been turned against the Universal Declaration of Human Rights.^55^

NAIRU economics was starting to manifest itself in laws on central bank goals, ‘workfare’ and a push toward ‘flexible’ labour markets. Written into the Bank of England Act 1998, and the Treaties underpinning the European Central Bank, was the goal of ‘price stability’ (not exactly ‘zero’ or ‘low inflation’). This seemed to be interpreted by bank economists as more important than ‘full employment’.^56^ In 1996, Janet Yellen, future Federal Reserve chair, wrote that NAIRU reflected ‘structural aspects of the economy’,^57^ while Bill Clinton’s economic advisers were calculating the unknowable natural unemployment rate: an endeavour in which they failed.^58^ More than this, USA and EU governments had started to place more duties and sanctions on unemployed people to search for jobs that did not exist. Everyone had to be ‘activated’ to get full employment,^59^ it seemed, except government itself.

This said, in 1997, Stephen Nickell admitted that ‘there is no evidence in our data that high labour standards overall have any impact on unemployment whatever’.^60^ But Richard Layard remained confident that government should keep cutting labour rights for ‘a flexible system of wage differentials. Nothing else will do the trick’.^61^ Economics papers were now written arguing that labour rights kill jobs.^62^ But even then, the better side of the evidence (and overwhelming evidence today) does not merely refute the notion that labour rights, especially employment protection, harm the economy. From the legal database of 117 countries, using econometric analysis at Cambridge’s Centre for Business Research, it appears that labour rights may advance employment, productivity, growth, equality and therefore human development.^63^ Labour rights also spur innovation.^64^

Critically, the original NAIRU argument (governments seeking full employment would accelerate inflation) was quietly segregated or forgotten.^65^ So were some hard facts. Even ignoring chronic under-employment and wage insecurity,^66^ unemployment was always a political choice, nowhere more so than in the USA.

This chart shows that from 1952 to 2020, Democrats always left the White House with unemployment lower: down 12% overall. Republicans always left the White House worse: up 14% overall, even without including Trump and COVID-19. The chart also calculates that, by including mass incarceration, the effective unemployment rate was at least 1.3% higher than the recorded rate in recent decades.^67^ Is the Republican record on jobs an accident? Probably not. In 2019, Donald Trump’s Chief of the Federal Reserve, a trained lawyer rather than an economist, stated that the link between inflation and unemployment was ‘weaker’ and a natural rate ‘lower than we thought’.^68^ The better view seems that a natural rate of unemployment was always an set of evidence-free conjectures, propped up by a chimerical hand.

### B. Technology

A second view of unemployment is it results from technology. In 1930, in *The Economic Possibilities of our Grandchildren*, John Maynard Keynes argued that society was being afflicted by ‘technological unemployment…due to our discovery of means of economising the use of labour outrunning the pace at which we can find new uses for labour’.^69^ He added that any unemployment from technology was ‘only a temporary phase of maladjustment’ and ‘mankind is solving its economic problem’. Keynes thought society could be four to eight times richer by 2030, and that we could endeavour to ‘make what work there is still to be done to be as widely shared as possible’ by having a 15-hour working week.^70^

Keynes was certainly not alone in seeing technology as a problem but also an opportunity. Joseph Schumpeter wrote in 1942 that new technologies were like a ‘perennial gale of creative destruction’.^71^ A ‘rapid change’ could disorganise industry and ‘create avoidable unemployment’. Yet we could, said Schumpeter, ‘avoid coming down with a crash’, and make an ‘orderly retreat’ instead.^72^ Most popularly, George Orwell in 1945 wrote in *Animal Farm* how the animals ‘listened in astonishment’ to how ‘fantastic machines…would do their work for them while they grazed at their ease in the fields or improved their minds with reading and conversation’.^73^ Once their human oppressors had been evicted, a future of leisure, a 3-day week, was possible through technology, and collective endeavour. In this way, Orwell, Schumpeter and Keynes all believed that technological change could have an impact upon jobs. Did technology mean more work, or more freedom? The answer, they said, depends on social policy.

In stark contrast, a view spread recently that ‘technological unemployment’ could be permanent. In 1995, in *The End of Work*, Jeremy Rifkin argued one of the great, unspoken tragedies of the American labour force had been mechanisation in agriculture.^74^ This (not absence of labour rights) led to mass post-war redundancy of farm workers in the south,^75^ and triggered Martin Luther King Jr’s ‘March on Washington for Jobs and Freedom’ in 1963.^76^ By the 1990s, computers had become like a terminator, ‘steadily moving up the office hierarchy, subsuming not only routine clerical tasks but even work traditionally performed by management’.^77^ The solution to unemployment, argued Rifkin, was a new value added tax in the USA to fund a ‘social wage’ for people to do volunteer work.^78^ In 2013, Erik Brynjolfsson and Andrew McAfee also argued that US unemployment, and workers’ stagnating incomes, result from ‘losing the race against the machine’.^79^ Government should educate and invest more, but continue ‘resisting efforts to regulate hiring and firing’.^80^ Drawing on Friedman, they advocated a ‘negative income tax’ where people in or out of work receive a basic income up to a limit to act as a ‘subsidy on labour’.^81^

What is truly surprising, especially given the authors’ social democratic views,^82^ is that the ‘ossification of American labour law’ is missing to explain US unemployment and inequality.^83^ In essence, since 1976, when a disastrous US Supreme Court case held the rich could spend unlimited money on political campaigns as part of ‘free speech’, there was an almost total halt in American social progress.^84^

**Figure 4.**
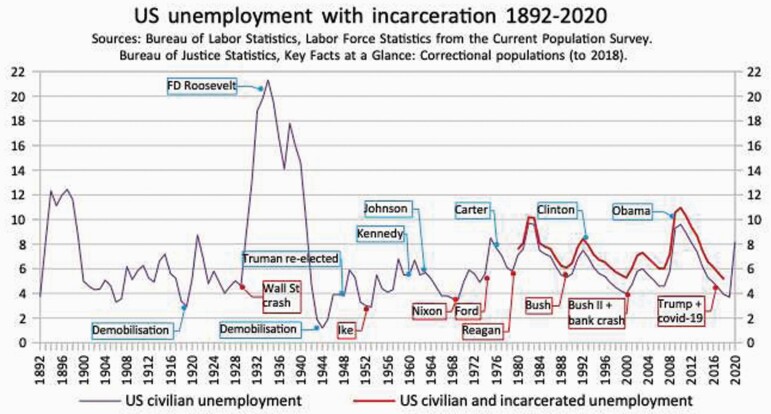
US union density and income inequality, 1930–2020. *Sources*: Bureau of Labor Statistics, 1930–74, http://unionstats.gsu.edu/All-Wage-and-Salary-Workers.htm. World Inequality Database, wid.world/data.

Figure 4 shows that as big money backed Reagan, labour was attacked, trade union density fell and inequality soared. The same pattern holds in systems where unions are the main channel for voice at work.^85^ In the USA, collective agreements are critical to regulate hiring and firing because there is just one federal Act to protect job security.^86^ This worsens unemployment. The easier it is for a conflicted, irrational authority figure to point a little finger and bark ‘you’re fired’, the worse is an economic crash.

**Figure 5.**
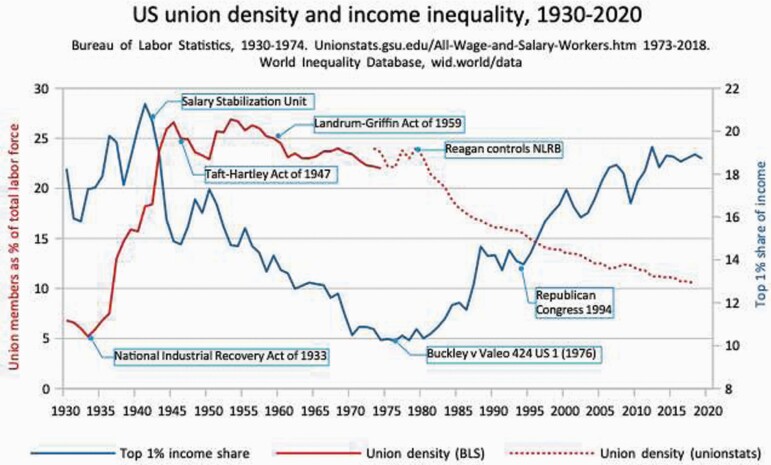
Unemployment, global financial crisis and COVID-19, 2006–20. *Source*: OECD, ‘Unemployment rate: Total % of labour force’. ONS, Unemployment.

Figure 5 shows the unemployment effects of the global financial crisis and the COVID-19 pandemic under three legal alternatives. In the USA, with employment ‘at will’, unemployment soared by 5.3% from May 2008 to October 2009, and again by 11.5% after the COVID-19 lockdowns in 2020. In the UK, employees must have reasonable notice, before a fair dismissal, and redundancy pay enforceable in a Tribunal.^87^ Unemployment rose 2.8% with the bank crash,^88^ and 1% with COVID-19. The latter, comparatively smaller rise is instructive because of the government’s ‘furlough’ and ‘job retention scheme’. In the two months after lockdowns the UK’s ‘claimant count’ for unemployment benefits rose by a staggering 2.1 million people, which would have meant over 10% unemployment.^89^ As this impending disaster became clear, it seems that tax authorities required that employers kept unemployment benefits claimants in their jobs subsidised in the furlough scheme. Ultimately, the unemployment statistics (not claimant count) showed just a 1% rise from February to October 2020. In Germany, there is much better notice and redundancy pay, but also employee elected work councils may veto or defer dismissals that are just individually or socially justified.^90^ Unemployment rose just 0.8% with the bank crash, and 0.9% with COVID-19, because German work councils negotiated working time reductions and pay restraint, including executives, to stop job losses.^91^ Furthermore, like the UK furlough scheme, Germany subsidises shortened working time *(Kurzarbeit*).^92^ In all countries there is more under-employment, making the unemployment statistics depart from Beveridge’s definition of full employment as with ‘fair wages’ and necessary hours.^93^ But the basic point is that if it is easy for conflicted, irrational managers to make people unemployed, there will be more, not less, unemployment.^94^ Econometric analysis at the Centre for Business Research shows that this is right. Job security improves employment, equality and prosperity.^95^

The narratives of Rifkin, Brynjolfsson and McAfee are less troubling than recent tech predictions. In 2013, Frey and Osborne argued that ‘47 per cent of total US employment’ was ‘potentially’ at risk from automation in a working paper abstract.^96^ They also claimed that inequality was due to technology, without mentioning changes in labour law.^97^ The paper was accepted into a peer reviewed journal in 2017, and by then the ‘47 per cent’ claim was cited across the media, and soon in academia. Few citations picked up that change would be ‘over some unspecified number of years’.^98^ Moreover their ‘method’ was that ‘with a group of [machine learning] researchers, we subjectively hand-labelled 70 occupations, assigning 1 if automatable, and 0 if not’ by ‘eyeballing’ different tasks.^99^ In 2015, Frey and Osborne secured funding from Citibank ‘Research’,^100^ and similar predictions went viral. These predictions were not based on any new data, but rather on a review of ‘research’ papers from McKinsey,^101^ PwC,^102^ Deloitte (working ‘with researchers at Oxford University’),^103^ and other consultancy businesses trying to boost demand for themselves by stoking social anxiety. More academics have learned the game. The Oxford ‘Future of Humanity Institute’ released a 2017 paper (part-funded by a Facebook billionaire) saying that robots will replace most human functions, and write a bestselling book by 2049. It reached this conclusion by surveying 352 ‘AI experts’.^104^

It is questionable whether technology predictions can be classed as normal academic enquiry. They have certainly proved controversial among academic economists,^105^ and the Obama White House.^106^ If the job of making predictions about future employment could be automated, what would the robots say? Would they reject the ‘research’ by McKinsey, PwC or Deloitte as little better than a crystal ball, guessing future stock prices or race horse winners? These predictions are highly vulnerable to conflicts of interest. But also, if mass job loss is predicted ‘over some unspecified number of years’, how can this really mean anything? For instance, from *The Canterbury Tales* in 1387, it would appear that there has been a mere 46% redundancy rate for Geoffrey Chaucer’s characters (Table 1). That is, in around 630 years, a rate one per cent under Frey and Osborne’s prediction. It might be objected this *Canterbury Tales* study has an unscientific method, uses a questionable categorisation of jobs, with lazy application of statistics. But if all this is true, it has one thing which the new methods of ‘research’ do not: evidence.

**Table 1.** The Canterbury Tales Study

Employed (1 point)	Redundant (0)	Part employed (0.5)
Chaucer (playwright)	Knight	Friar
Host (hotel owner)	Miller	Ploughman
Wife of Bath	Reeve	Prioress
Cook	Summoner	Monk
Sergeant of Law	Squire	Nun’s priest
Clerk	Franklin	Second Nun
Merchant	Pardoner	Parson
Physician	Canon Yeoman	
Shipman		
Manciple (caterer)	**13 of 25 employed**	**or 46% redundant**

### C. Law + Capital Supply

A third main view is that, far from being natural or technological, unemployment results when law enables the restriction of capital supplied to the job market. Capital, said Adam Smith, is the part of one’s stock that is used to make ‘revenue’, but is not for ‘immediate consumption’.^107^ That is, capital is property used for production.^108^ This insight drove New Deal and post-war UK government policy.

First, as the US depression reached its height, John Maynard Keynes became the great theorist of its policies.^109^ Keynes argued that most unemployment could not be considered as voluntary.^110^ If total spending or effective ‘aggregate demand’ for goods, services and labour dropped away, involuntary unemployment results.^111^ Poorer people have a higher ‘propensity to consume’ their income, because they need to spend everything as they get it. Wealthier people save, or invest in the financial sector.^112^ Before the Wall Street Crash, more and more affluent Americans invested in the stock market for retirement and, said Keynes, the ‘mass psychology of a large number of…individuals is liable to change violently’.^113^ Even skilled business people have an ‘extreme precariousness’ in their ‘basis of knowledge’ to calculate investment returns in future.^114^ Triggered by speculative swings, cycles of boom and bust result when business over-invests in productive capacity, stock piles up, production is cut back, and business makes people redundant. Most critically, poor ‘social practices and…a distribution of wealth…result in a propensity to consume which is unduly low’.^115^ This slows the ‘velocity of money’ flowing through the economy,^116^ and with it aggregate demand. Asset owners cannot be relied upon to boost aggregate demand in a depression, because they have a lower propensity to consume. So, active government should balance business volatility and irrational thrift.

Keynes was careful to say that we ‘should not conclude…everything depends on waves of irrational psychology’.^117^ But the role of legal policy was not as prominent for Keynes as it was for other New Dealers. For A.A. Berle, a corporate finance lawyer and author of Roosevelt’s famous speech arguing for ‘an economic declaration of rights, an economic constitutional order’,^118^ it was clear that the Supreme Court’s crushing of social security had led so many Americans to invest in the stock market.^119^ It was clear that these people, without protective regulation in securities markets, had then become prey for promoters, speculators and bankers, who dealt in ‘other people’s money’ and led the Wall Street crash.^120^ The Social Security Act of 1935, as the US Supreme Court reversed its ‘freedom of contract’ case law,^121^ ensured everyone had the right to an old age pension and unemployment insurance.^122^ The Securities Act of 1933 and the Securities and Exchange Act of 1934 mandated disclosure for all securities contracts, forbid conflicts of interest, and required public enforcement. Correcting these problems was basic to guarding against massive crashes and resulting unemployment in future. Indeed, the global financial crisis in 2007 was driven by the Bush II administration eviscerating federal securities regulators,^123^ and consumer protection,^124^ which enabled speculation on people’s right to a home.

Second, in 1951 according to M.S. Eccles, Chair of the Federal Reserve, Wall Street crashed and depression had ensued because wealth accumulated as ‘idle or hoarded funds’. Speculation and consumer debt maintained production and jobs for a while. But like ‘a poker game where the chips were concentrated in fewer and fewer hands’, when most people’s ‘credit ran out, the game stopped’.^125^ If there had ‘been a better distribution of the current income’ with ‘lower prices or higher wages and with less profits to the corporations and the well-to-do, it would have prevented or greatly moderated the economic collapse’.^126^ Property carries responsibility,^127^ and people holding productive property had more.

Third, the 1944 UK White Paper, *Employment Policy* saw swings in investment as the barrier to full employment. Consumer spending is relatively stable, and government could rationalise its spending. But private and international investment were volatile. Government should balance both, shifting projected spending forward or back in five year terms.^128^ At the same time, large employers acquired duties to hire disabled people up to a quota,^129^ and government ensured investment into under-developed regions, so the country did not languish behind cities.^130^ The Board of Trade would create development areas if ‘there is or is likely to be a special danger of unemployment in that area’.^131^ John Wardlaw-Milne, a backbench MP, proclaimed this was the ‘very antithesis of private enterprise…bureaucracy and Socialism carried to the last limit’.^132^ And with Conservative, Labour and Liberal consensus, it worked for 30 years.

The theory behind full employment was not all that new. In 1912, Sidney Webb had advocated government spending to counteract shifts in foreign trade and private consumption, public labour exchanges to match workers to job vacancies efficiently, for people to be guaranteed a minimum number of hours a week instead of being ‘on call’ (with ‘zero hour contracts’), and progressive reduction of everyone’s hours of labour over the long term.^133^ For the relatively few ‘won’t works’, those people should be given training, support and discipline, but not crime and punishment.^134^ By contrast, in 1943 Michal Kalecki argued that discipline was the real reason unemployment existed. If there were full employment, ‘workers would “get out of hand” and the “captains of industry” would be anxious to “teach them a lesson”’.^135^ Employers have an incentive to restrict investment in jobs, to reduce worker bargaining power. If unemployment is high, employers as a group have few reasons to do anything to reduce it.^136^

But after World War Two (WW2), full employment *was* maintained by political consensus in the UK, as it was in Australia, Japan or Germany.^137^ In 1962, A.A. Berle added that a legal duty for full employment could extend to corporations. Just as government would be responsible to maintain spending, companies ‘ought not to hoard when expenditure is needed’ and they might need ‘to slow down under some conditions’.^138^ The most fascinating fact was that full employment cost government very little. According to Robin Matthews, far from a ‘Keynesian revolution’ with counter-cyclically massive government spending, the UK government post-war had a constant current account surplus. This indicated spending restraint.^139^ In other words, once government had made the initial investments, and then committed to do ‘whatever it takes’, this maintained business confidence, economic stability and full employment.^140^ After the OPEC price hikes, once confidence resumed, full employment would have been possible again. Even if jobs *are* paid for, the COVID-19 pandemic illustrates the low cost once more. By October 2020, under the UK government’s Coronavirus Job Retention Scheme, just £43 billion was needed to pay 80% of the wages for 8.8 million people at its peak, about 5% of the UK annual budget.^141^

The weakness in theories that have emphasised law’s role in the supply of capital for jobs is the prominence given to government to guarantee investment. Kalecki already feared that a government hostile to the effect of full employment on labour relations would scrap it all.^142^ Why should democratic society expose its prosperity to interest groups that would undermine its very basis? After OPEC, full employment was abandoned. An era of recurrent crisis took hold. The financial sector inflated. Corporations began hoarding more assets.^143^ Economic theories were chosen to justify it. The return of corporate cash hoarding since the 1990s has been nothing less than extraordinary,^144^ and nowhere more than among tech-corporations. By 2016, US non-financial firms held $1.68 trillion in cash, $1.2 trillion overseas. Apple, Microsoft, Alphabet, Cisco and Oracle alone held $504 billion in cash.^145^ That money, $504 billion hoarded by just five tech companies, was enough in 2017 to give every unemployed person in the USA a full-time job on $10 an hour for a four year presidential term.^146^ This is the socially irrational product of what seem to be individually rational firm decisions to save. It challenges the legitimacy of the modern corporation and private property as it is,^147^ because it damages human development. Unemployment is caused by law, not individuals or their technology. Society is able to change it.

## 3. TECHNOLOGY AND JOBS: FIVE LESSONS FROM HISTORY

We cannot know the future of technology’s impact on jobs, but we can learn from the past. The best lessons for what to do are times of significant historical change. Five are most instructive: (1) the shifts from agriculture to wool and mechanised textiles, (2) the massive post-war demobilisations, (3) the impact of motor vehicles on horses, (4) the washing machine and (5) computerisation and AI. History shows that technology improves lives, if people organise thoughtful social policy in response.

### A. Sheep + Luddites

A first lesson starts with the displacement of agricultural labour in Reformation England. It shows how people react if they cannot share in technology’s gains. By 1516, a disruptive, even devastating ‘technology’ had multiplied the value of land as never seen before. ‘Sheep’, wrote Sir Thomas More,^148^

have now apparently developed a raging appetite, and turned into man-eaters….Each greedy individual preys on his native land like a malignant growth, absorbing field after field, and enclosing thousands of acres with a single fence. Result—hundreds of farmers are evicted. They’re either cheated or bullied into giving up their property, or systematically ill-treated until they’re finally forced to sell…men and women, husbands and wives, widows and orphans, mothers and tiny children….you can’t run a farm without plenty of manpower.

The sheep—or more specifically, wool—market, wrote More, was ‘almost entirely under the control of a few rich men, who don’t need to sell unless they feel like it, and never do feel like it until they can get the price they want’. Worse, this led to a ‘great army of unemployed’, and turned people ‘into beggars or thieves’.^149^ More recommended reviving employment for ‘plenty of honest and useful work’.^150^ But the response of the state was ever stiffer penalties. Under the Vagabonds Act 1530, it was decided that unjustified begging should be punished with a good whipping. This did not seem to work, and meanwhile in 1535 More was executed. So, the Vagrancy Act 1547 placed beggars in servitude, or slavery for a second ‘offence’. Kett’s Rebellion in 1549 led to a repeal,^151^ but criminalisation of the poor and enclosure went on.

With wool market expansion, in 1589 an inventor named William Lee created the first mechanical knitting machine. Queen Elizabeth I refused Lee a patent, fearing stocking workers would become redundant. She asked him to invent something for silk instead.^152^ Whether this slowed mechanisation or not, steam power sped development up. In 1769 Samuel Wise, a clockmaker, attached a cog to stocking machines that turned with steam.^153^ Steam and machine meant unprecedented speed. Workers were made redundant, and in 1779 an apprentice named Ned Ludd was said to have smashed machines in Leicester after being whipped for vagrancy. Even if this was a myth, Ludd became legend, as sporadic ‘Luddite’ and ‘swing’ riots broke out. The Protection of Stocking Frames, etc. Act 1788 section 4 required 7 to 14 years’ transportation for destroying machines, just as the ‘First’ Fleet of Arthur Phillips anchored in Australia. The Destruction of Stocking Frames, etc. Act 1812 imposed a death penalty.^154^ Lord Byron opposed it in his maiden speech.^155^ The machines ‘superseded the necessity of employing’ workmen, said Byron, ‘who were left in consequence to starve’. ‘It is the mob that labour in your fields, and serve in your houses…and can also deny you, when neglect and calamity have driven them to despair’.^156^

**Figure 6.**
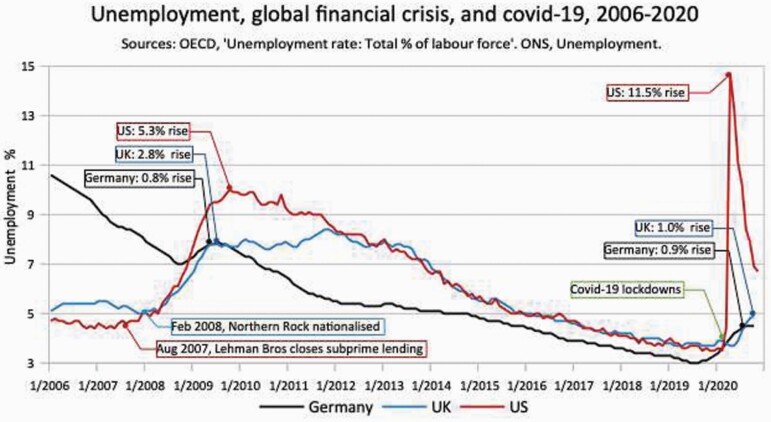
Real wages in England 1700–1869. *Source*: G. Clark, ‘The Long March of History’ (2005) WP UC Davis No 05, 40.

Unrest continued for decades, as real wages stalled and fell. It was not merely that people’s incomes convulsed with the wild paroxysms of agricultural prices. Especially from 1747, and again from 1795, long-term down-swings in wages were punishing, recurring until the labour movement formed (Figure 6).^157^

Did wages decline because of technology in the Industrial Revolution? No. Law caused the decline. First, the Master and Servant Act 1747 wrought a massive extension to all classes of workers of punishments for leaving employment, and for ‘justices of the peace’ to cap, fix and repress wages.^158^ There was also no right to unionise and strike,^159^ but employers could form partnerships, corporations, and dismiss workers regardless of the social cost.^160^ Second, on 6 May 1795, a meeting of poor law magistrates at a ‘Pelican Inn’, in Speenhamland, Berkshire, decided unemployed people, *and* the working poor, should get relief because grain prices had risen (again).^161^ They rejected a minimum wage.^162^ This reckless, paternalist act was a cue for employers to use parish subsidies as an excuse to cut employment wages. It was not merely that people in work became dependent on poor law relief.^163^ Nor is it relevant that Speenhamland was replaced by an even ghastlier system of workhouses, based on concocted evidence, in the Poor Law Amendment Act 1834. As working people had no vote in politics,^164^ this ‘basic income’ only lined landlords’ pockets, the very ‘definition of a rentier’ system.^165^ It made, wrote Eric Hobsbawm, ‘universal pauperism of demoralised men who could not fall below the relief scale whatever they did, who could not rise above it…reduced to as little as the village rich thought fit for a labourer’.^166^

Slow change began in 1834, when a group of agricultural labourers in Tolpuddle, Dorset, were sentenced to transportation for making ‘unlawful oaths’. They had formed a union.^167^ They did not break machinery but, wrote Sidney and Beatrice Webb, their trial followed the ‘specially hard times of 1829…outbursts of machine-breaking, rick-burning and hunger riots…put down in 1830 by the movement of troops…1000 prisoners, several of whom were hung and hundreds transported’.^168^ As the labour movement rallied in their support, they became the ‘Tolpuddle martyrs’, all because the government insisted on making an example of them. Far more than Luddites, this became the real legend of the labour movement. It was not until the Trade Union Act 1871 that unions finally became lawful, and not till the *Taff Vale* case, the formation and electoral success of the Labour Party, and the Trade Disputes Act 1906, that the right to collective action was secure. But this episode of history shows, when they do not share in the benefits of technology, people revolt and resist. Eventually, they organise.

### B. War + Demobilisation

A second lesson of history comes from demobilisation after WW1 and WW2. Unlike most wars of the 18th and 19th centuries, total war, with conscription, employed a large minority of the population directly. As the chart in part 2(A) showed, demobilisation in the UK after WW1 led to soaring unemployment, near 18% by 1922. The norm lurched above 10% until the rearmament for WW2. It was chaos, with all the ‘dimensions of a calamity’ and British labour fought before it accepted ‘a reduction of its standard of living’.^169^ While David Lloyd George had promised ‘homes fit for heroes’, his post-war coalition had no credible plan for jobs.^170^ After Armistice, 30,000 workers on Tyneside marched against unemployment, 3,000 protested rapid demobilisation on New Year’s Day of 1919 in the Rhondda Valley, and 50,000 iron workers and engineers struck in the autumn. Between 1920 and 1931, over twenty Acts were introduced to Parliament to tinker with unemployment policy.^171^ But even after the General Strike of 1926, responding to coal miner wage cuts of 43%, none bit the bullet: the need for law to guarantee full employment.

**Figure 7.**
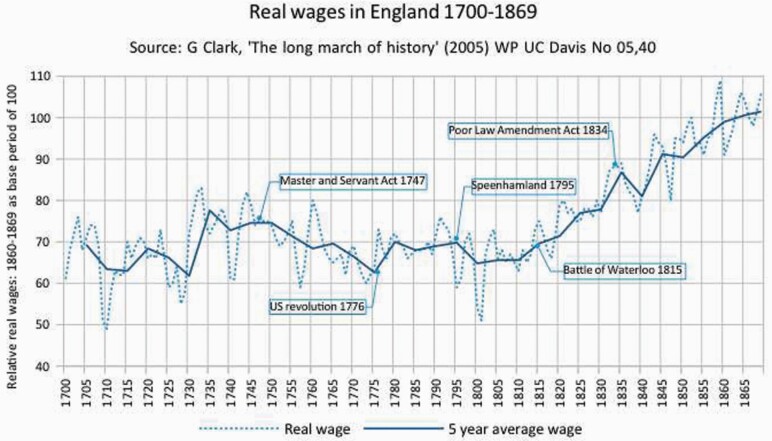
US unemployment with incarceration, 1892–2020. *Sources*: Bureau of Labor Statistics, Labor Force Statistics from the current population survey. Bureau of Justice Statistics, ‘Key Facts at a Glance: Correctional Populations (to 2018).

**Figure 8.**
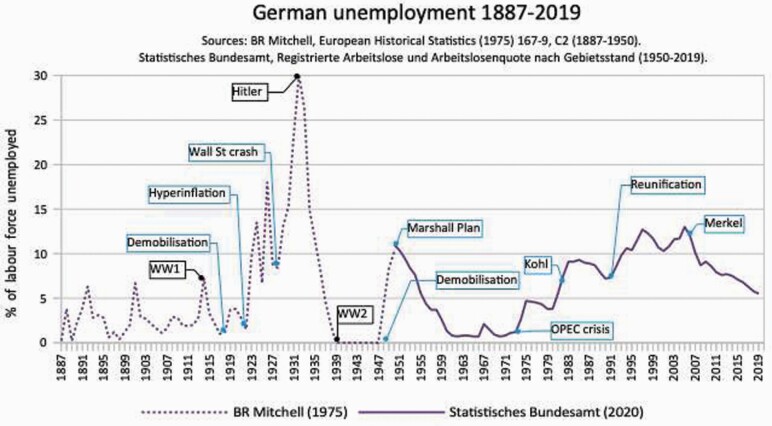
German unemployment 1887–2019. *Source*: B.R. Mitchell, European Historical Statistics (1965), 167–9; C2 (1887–1950). Statistisches Bundesamt, Registrierte Arbeitslose, Arbeitslosenquote nach Gebietsstand.

The United States fared better, partly because of its sheer size and shorter involvement in the war, partly because a million men were employed on public works in 1919. But as Figure 7 on US unemployment shows, it did not save the Democratic Party’s hold on the White House.^172^ One might expect that Germany fared worse: 6 million soldiers redundant upon the Reich’s collapse, 800 thousand people returning from captured territory,^173^ its economy in ruins, its political class in infamy. Yet against the UK and USA, the immediate rise in post-WW1 unemployment was the least (Figure 8).

This was because Social Democrats passed laws preventing dismissal without good reason, involving unions and elected work councils, and organised labour exchanges.^174^ It all changed, of course, with the erection of tariff barriers by US President Harding,^175^ with the malevolent terms of the Versailles Treaty,^176^ the resulting inflation, and then the government’s crackpot policy of hyperinflation that wrecked the German mark.^177^ The Germans’ roaring twenties meant endless humiliation, until a sociopath with a silly moustache promised respect. By 1939, as the Nazi state launched its campaign to enslave Europe it was financially bankrupt. Nothing could have been further from full employment in a free society.^178^

After WW2, both UK and US governments would not allow a repeat. As part 2(C) explained, the UK government’s White Paper, *Employment Policy* of 1944 committed to full employment. In June 1945, the UK had total workforce of 21.5 million men and women, with 5.219 million in the military, and 3.837 million producing for the military.^179^ That is, over 42% of the total workforce produced directly for war, and so were redundant on war’s end. The need to redeploy people was obvious: for ‘houses to be built, shops to be filled, factories to be transformed, plant and rolling-stock to be replaced and export trade to be renewed and extended’.^180^ Even if it was ‘vain to imagine that patches of unemployment can be eliminated altogether in the transition’ the White Paper said they ‘should not be so widespread or so persistent’. Government would switch capacity, arrange civilian work and products, and dispose of surplus government stock to avoid disrupting trade.^181^ This was the basis of economic recovery.

The counterpart plan in the USA involved three major Acts. First, the Servicemen’s Readjustment Act of 1944, or the ‘GI Bill’, funded retraining and education fees for veterans.^182^ Second, Congress passed the Employment Act of 1946 to ‘promote maximum employment, production, and purchasing power’.^183^ Once passed, the Truman government influenced fiscal and monetary policy to grow employment, even if Eisenhower would refuse to follow. Third, the Foreign Assistance Act of 1948 led to $12.7 billion in grants and loans for Western Europe. According to this ‘Marshall Plan’, the assistance was to ‘provide a cure rather than a mere palliative’ for ‘restoring the confidence of the people of Europe in the economic future’.^184^ The results were tremendous: a new era of European and American prosperity.

The real lesson from demobilisation is that, with concerted action, even the massive shock of 42% unemployment in the UK, and similar figures elsewhere, were swiftly contained. Demobilisation was an infinitely greater challenge, in size and social complexity, than any credible prediction about the effect of automation. Hundreds of thousands of people were disabled as a result of war, physically and psychologically. Entire economies had to shift, not over decades as technology was rolled out, but at the instant of armistice, to ‘win the peace’. There was nothing that labour law, dismissal protection, redeployment rights, and full employment, could not accomplish. As the Universal Declaration said, this was the true meaning of the ‘right to work’.^185^

### C. Smoke + Horses

Although social policy can ensure massive shocks to employment are contained, a third lesson of history is that technology’s replacement of labour, even if total, can be slow. In 1885, Gottlieb Daimler patented an internal combustion engine for a motorbike. In 1886, Karl Benz patented the first motor car in Germany. Their engines ran on petroleum gasoline, refined from oil, a resource whose mass extraction was equally recent.^186^ Mass motor production only began in 1908, with the Model T Ford. Motor vehicles, cars and trucks, did not merely compete with steam engines and rail: those could not reach specific delivery points. No, this technology meant the horses of the world were soon out of a job.

Many could not accept the inevitable. In 1908, Mr H.B. Brown wrote in the *Yale Law Journal* that for ‘park and other pleasure driving’ when cars ‘cease to be a fashionable fad, the public will probably return to carriage and horses’.^187^ This was, said Brown, because cars were ‘attended by a cloud of dust and smoke’, ‘the emission of a noisome odor’ and the ‘cold and heartless mechanism of the automobile’ was to many a ‘veritable terror’. Indeed, the ‘automobile lacks one of the most attractive concomitants of pleasure driving in the companionship of the horse’.^188^ Brown urged his readers to ‘pray’ against ‘the extinction or dethronement of the noblest of all domestic animals’. But his prayers would be brutally run down. Relentless, unforgiving, the car stomped the US population of horses from 26 million in 1915, down to 19 million by 1930 and 3 million by 1960. Mass horse unemployment became a reality: 88% of horse jobs galloped away.^189^

**Figure 9.**
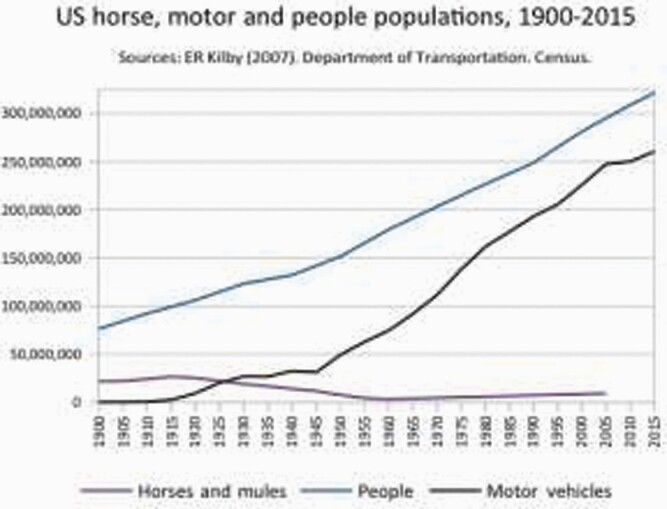
US horse, motor and people populations, 1900–2015. *Source*: E.R. Kilby. Department of Transportation Census.

Yet the lesson is that, even though a new technology made an old one obsolete, the transition with matters left mainly to the market took 45 years (Figure 9). Factors slowing transition include the lifespan of horses, the costs of switching once horses were bought, the costs of upgrading related infrastructure networks (e.g. stables to petrol stations), and no doubt elements of romantic attachment. What could this mean for drivers of cars and trucks? If driverless technology becomes real and safe for commercial use, redundancies may be very slow.

**Figure 10.**
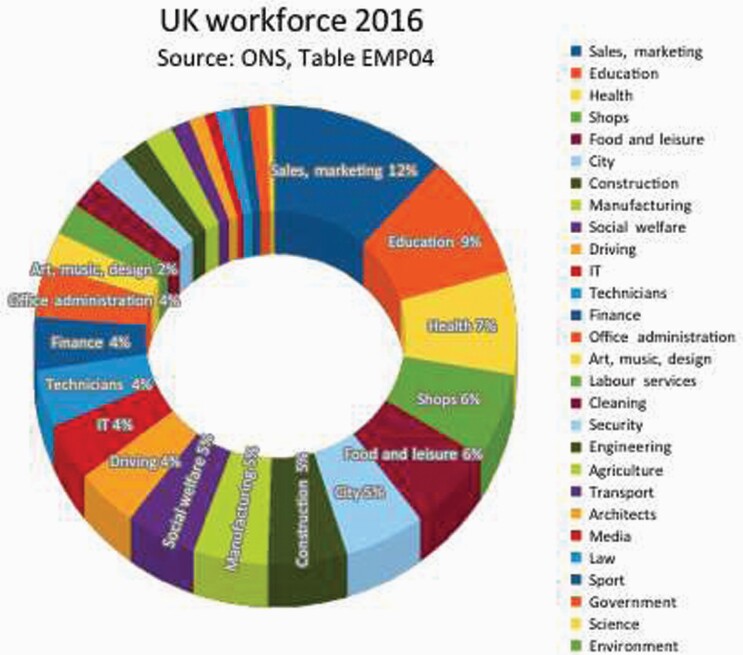
UK force 2016. *Source*: ONS, Table EMP04.

Driverless vehicles and jobless drivers have become a central image in automation discourse. So it is worth asking, will people be as jobless with robots, as horses were jobless with cars? The answer is almost certainly ‘no’. First, as Figure 10 above shows, the actual proportion of driving jobs is relatively modest: taxis, public transport, delivery and on-site motors. In 2016, it was near 4% of the UK workforce, just under 1.3 million people. Second, many commercial aeroplanes are computerised, and there is still a need for a pilot, not merely to supervise the robot, but to change course, take-off and land most of the time. Much the same is true for trains. Even in supermarkets, which have partly ‘automated’ checkouts (and quietly turned customers into workers^190^), staff monitor the machines’ constant malfunctions. But third, even if all robotic problems are overcome, it is doubtful that human beings will become redundant like horses. Human beings not only enjoy the ‘companionship’ of other humans, including in ‘customer service’. Humans, not horses, write laws and can protect their own welfare. Unless some people manage to treat others as disposable commodities or beasts, jobs can be improved.

Rapid transition to automated work is both desirable and necessary. For example, in 2015 the deaths from avoidable traffic collisions totalled 84,589 in Europe, 34,064 in the USA, 238,562 in India, 261,367 in China, and 1.25 million across the world.^191^ Vehicles driven by humans are unsafe at any speed. Everything must be done to end the misery, and secure everyone’s freedom from harm on the roads.^192^ Second, many drivers suffer from stress, loneliness and monotony.^193^ Automation is an opportunity to humanise work, if drivers are redeployed with an income guarantee. Third, if driverless vehicles can be introduced, with no salaries to pay, it will radically reduce the cost of production. The gains can be made green: every ‘no driver’ vehicle could be a ‘no smoking’ vehicle too. But the transition to a safe, zero carbon transport system will only happen with foresight and smart regulation, not by ‘spontaneous order’.

### D. Washing Machines

History’s fourth lesson is technology drives human development most when people internalise the gains. If motor vehicles were a significant technology rolling out before WW1, the washing machine was the equivalent rolling around before WW2. In 2010, Hans Rosling described the washing machine as being like ‘magic’, a ‘miracle’ for human development.^194^ Before, all laundry was done by hand, labour usually without pay, and it was seen as a woman’s job. Washing history reveals how all-consuming the issue was. In 1841, Catharine Beecher and Harriet Beecher Stowe exclaimed: ‘How would it simplify the burdens of the American housekeeper to have washing and ironing day expunged from her calendar!’^195^ Without the technology there, they called for a common system of laundries, to save on individual labour.

**Figure 11.**
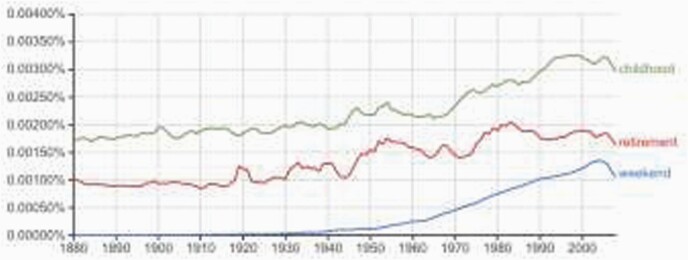
Childhood, retirement, and weekend word use: Ngram.

Even though washing machines began to be mass produced in the 1940s, distribution took time, and remains a question of development. From the 1950s, Katharine French-Fuller recounted how in Chile ‘every week all year long—every week without fail—there was washday….The clothes would be scrubbed…by a woman bending over the tub’.^196^ In 1985, Jean Robinson recorded a Chinese woman saying: ‘It’s really a pain not to have plumbing. Every day we need about 4 or 5 buckets of water…every day it takes more than hour just to get the water, and once we’ve used it, I have to get rid of it’.^197^ In other words, from Chile to China, with access to plumbing and automated washing, women could save a whole day’s worth of work each week. The washing machine was almost as powerful as collective bargaining from the 1920s and 1930s, which not only spread ‘childhood’ and ‘retirement’ for most people,^198^ but (as Figure 11 shows^199^) actually created the word of a two day ‘weekend’.^200^ By 2010 Rosling estimated that 2 billion people had access to a washing machine, though 5 billion people still did not.^201^

To advance gender equality,^202^ and save working time, technology like washing machines needs public infrastructure: a piped water network, and an electricity grid.^203^ In 2015, the World Bank recorded that just 71% of the world’s population used ‘safely managed drinking water services’, a figure stagnant since 2010, and one which does not necessarily mean that water is piped and pumped. In 2014, 85% of people had access to electricity. Road infrastructure is needed for private transport, just like as public communication networks enable giant websites to function.^204^ Water and electricity networks are the same for washing machines. For automation itself, a wider ‘network’ of institutions is needed, particularly higher investment.^205^ When everyone can share in technology’s gains, when people can internalise their gains through distributed benefits, human development will be rapid. Social organisation makes it happen.

### E. Artificial ‘Intelligence’

A fifth lesson of history is also one of the future. Science fiction is filled with optimistic and apocalyptic predictions. In 1989, *Back to the Future: Part II* said that we would see a flying skateboard by 21 October 2015. Of course, this was fiction. But how different is the marketing hype on hyperloops or ‘personal air vehicles’? Similarly, in 1991, a James Cameron film projected on 29 August 1997 computers would become self-aware, launch a nuclear war against human beings and develop a robot army to eradicate the rest.^206^ Again, this is fiction. But how different was Rifkin’s apocalyptic *End of Work*? Indeed, the net effect of computers appears to have been an increase in employment, not an ‘end’.^207^ The same appears true for robots and manufacturing employment in US metropolitan areas.^208^ The lesson is, while the only limit of imagination is itself, the limits of physical science follow another script.

When it comes to the replaceability of human labour, particularly cognitive work, it is true that computational power exceeds human beings in strength and speed. However, it is important to distinguish the various euphemisms behind ‘machine learning’, ‘deep learning’ and ‘artificial intelligence’. ‘Machine learning’ is ‘the properties of an algorithm—a series of mathematical instructions’ in software that enables parameters to change based on repeated exposure to data ‘without being explicitly programmed’.^209^ Of course, the first step is programmed, before the computer continues, like you control the direction when you first skim a stone over waves in the sea. ‘Deep learning’ assigns weights to different kinds of data, and using a technique called ‘convolution’ may recognise images that are similar, but slightly varied.^210^ This is fascinating, but anyone who has been asked to confirm ‘I am not a robot’ (by distinguishing traffic lights, vehicles or pavements) is generating the actual human data (and doing unpaid work for Google’s subsidiary reCaptcha) that goes into the computer. Deep learning also continues to struggle with natural language processing, as artificial ‘neural’ networks ‘lack the capacity to produce decomposable abstractions and ontologies’.^211^ Why did the computer cross the road? It may never know.^212^

A computer is not learning. It is computing.^213^ Real ‘intelligence’ means ‘knowledge’.^214^ Data is not knowledge. Human beings have cognitive power, and that means ‘understanding’.^215^ Doing is not understanding. Human beings can interpret rules because they understand their meaning and purpose. Following rules is not interpreting them. Computers might process regulations, but is it possible to programme a sense of equity, let alone justice?^216^ In a democratic society, justice cannot be reduced to an arithmetic or geometric equation,^217^ because the parameters of society’s norms are recreated by the very act of interpretation. Can that be programmed? Computers might play and win games like chess or go.^218^ But as every teacher knows, rote-learning is not critical thinking. Human intelligence derives from at least five organic senses. It is completely unclear that a set of ‘0’s and ‘1’s, or even quantum computing, could logically be a basis for Cartesian self-awareness.^219^

While it is desirable that ‘machine learning’ develops, the philosophy of language shows why AI may not achieve the unique qualities of the human mind. With Bertrand Russell, one of the greatest philosophers of the 20th century, Ludwig Wittgenstein, believed language could be reduced to its ‘atomic’ components. Once the underlying logical form of any statement was analysed, they thought, its truth or falsehood could be ascertained.^220^ This logical positivism was heralded as an end of philosophy because it was thought philosophical problems were terminological misunderstandings that could be positively analysed, clarified and resolved. After WW1, Wittgenstein taught maths to primary school children, and became the fiercest critic of his earlier theory. Language had no underlying, logical structure because the meaning of a word is its use.^221^ Words have no literal or ‘original’ meaning because meaning exists through its context.^222^ Meaning is shared. Human intelligence is a social phenomenon. ‘Artificial’ intelligence is literally that: fake intelligence, not real. This is not a cause for pessimism, but instead should focus attention on what really matters. This is that technology’s true value is to accentuate and empower the uniqueness of the human mind, to help us to use it, and to help one another.

## 4. HOW TO REPROGRAMME THE LAW

While history shows that technology can improve jobs and lives with the right social policy, ‘technological redundancy’ (if not technological unemployment) does exist. This leads to the essential question: does the law sufficiently guarantee full employment on fair incomes, not mass unemployment on basic incomes? The Universal Declaration of Human Rights article 27 also enshrines the right of everyone ‘to share in scientific advancement and its benefits’.^223^ Does the law protect these rights? The short answer is ‘not yet’, but as technology develops, we can ‘reprogramme’ the law, to make the future of work and leisure compatible with human rights.

Before expanding, the oft-forgotten right to share in science’s benefits needs a brief explanation. According to an expert group convened by UNESCO in 2009, ‘this right is inextricably linked…to other rights, such as to a clean environment, education, information, labour rights, social security, sustainable development, water, where access to science is an implicit requirement for their full enjoyment’. Member states have positive obligations to ‘adopt a legal and policy framework to establish institutions to promote the development and diffusion of science and technology in a manner consistent with fundamental human rights’.^224^ The historical background to this right is key. With experience of the slow pace in electrifying rural America, and distributing a polio vaccine, the framers of the Universal Declaration realised ‘that access to essential determinants of quality of life—from electricity to vaccines to books…was crucially shaped by law and policy’. If electrification or health were ‘not to be a privilege of the wealthy few, as in the past, but a right to be assured to all’, the law had to prevent patents and corporate owners enclosing these goods from the public.^225^ The importance of distribution has also been recognised in economics discourse, in the Inequality-Adjusted Human Development Index. This discounts countries’ apparent development if gains in economic growth, education, and health, are not shared in practice.^226^

To address the distribution of technology’s gains, including the impact on work, there are currently three main groups of proposal. First, Richard Freeman and others argue we should enable more people to ‘own the robots’, and argues that more employee share schemes are the way to do this.^227^ Second, many authors, and even the entire 2020 US presidential campaign of Andrew Yang, advocate a basic income. This is often connected to a Scepticism of labour rights and social security systems.^228^ Third, many including the previous Obama White House advocate modest steps of expanding retraining, adult education or severance pay to deal with job-displacement from technology.^229^ The difficulty is, these proposals fall short of the baseline of labour and social rights in international law. By contrast, human rights often fail to articulate which policy mechanisms are needed to realise the rights. Accordingly, to reprogramme the law it is essential (1) to assure fair wages by democratising the economy, (2) to universalise social security by filling all gaps in the welfare system and (3) to guarantee a right to work on reduced working time by taxation on the hoarding of corporate profits.

### A. Economic Democracy

The rights to ‘just and favourable remuneration’, and to ‘share in scientific advancement and its benefits’ are enshrined in international law, but the mechanisms to achieve them are not. Part of the process is the right to join trade unions and take action,^230^ although collective bargaining and action do not by themselves guarantee outcomes. This is all the more true given the inherent inequality of bargaining power between labour, and employers who own capital, usually organised in corporate form.^231^

The basic problem is that within the corporate form, votes for corporate directors are held mostly by shareholders. In turn, it is crucial to understand that shareholder voting rights are monopolised by asset managers and banks, following the decline of individual share ownership, and the rise of institutional owners. Firms in the City, La Défense, Frankfurt or Wall Street invest money on behalf of people mostly saving for retirement, usually workers saving in pensions, life insurance or mutual funds. But these ultimate investors of capital are excluded from meaningful influence. For instance, in the USA, the three largest asset manager firms—BlackRock, State Street and Vanguard—if combined would be the largest shareholder in 438 of the S&P 500 largest corporations.^232^ In each firm, there are 10–20 people working in corporate governance departments, casting votes on corporate shares. This means that under 50 people control the weight of votes in the American economy.^233^ These financial institutions systematically oppose unions and fair wages, support escalating director pay, oppose action to stop discrimination at work and the gender pay gap, and oppose meaningful action to combat climate damage.^234^ But all their voting power comes from other people’s labour and ‘other people’s money’.^235^ In effect, workers’ capital is used against workers. As financial capital’s power over the votes in the economy augmented, labour’s share of income declined, and returns to capital exceeded growth.^236^ This means the benefits of scientific advancement, technology and automation will not be shared without giving the votes in the economy back to those who pay for them.

Economic democracy means the right to vote, along with the right of people to unionise, collectively bargain and take action on a sectoral basis in relation to all employing entities.^237^ The crowning achievements of collective bargaining have always involved transforming the employer’s internal governance: winning seats on company boards,^238^ pension trust boards^239^ and democratising public enterprises, such as universities, health trusts, transport, energy or water.^240^ Critically, the evidence shows that when people have a right to vote in their workplaces, workplaces are more equal, productive, innovative and prosperous.^241^ The right to vote at work may mean workers voting for a fixed number of directors on a board, having a share of votes in company general meetings, or both.^242^ The right to vote in capital means that workers and savers elect those who manage their money, and that intermediary asset managers or banks are banned from voting without instructions: Switzerland did this in 2013.^243^ When enterprises are in the public or regulated sector because markets fail to uphold the public interest, and we see people not merely as ‘consumers’ but members (like students, patients, passengers or ratepayers), a long tradition of law gives votes and voice to those members of the public.^244^

Economic democracy, with votes at work, votes in capita and votes in public services, changes investment and distribution decisions. When company directors account to working people, this limits the incentive to restrict investment in jobs and hoard capital. With votes at work,^245^ directors will stop paying themselves more and everyone else less.^246^ With votes in capital, asset managers and banks will be unable to inflate their fees with ‘other people’s money’.^247^ With votes as a member of the public in regulated services, people will no longer be exploited for their private data, held hostage to a train timetable or have to accept polluting electricity and vehicles. People will use votes to favour full employment, and a more just, sustainable society, expressing similar preferences to those they express in politics. This in no way obviates the need for democratic politics, as the COVID-19 pandemic and climate damage crisis vividly underlines the need for a ‘green recovery’.^248^ Democracy is universal, for every social institution.

An alternative proposal of Richard Freeman and others is employee share schemes. This takes the view that if we ‘own the robots’ we could live in a more equal society, and the risk of mass unemployment would go. Freeman argues for ‘employee ownership’ through the mechanism of trusts, stock options and profit sharing schemes, which is said to be like John Lewis, Mondragon or Google.^249^ The first major problem is that share schemes are often a form of payment in kind, or ‘truck’, not money. When workers are paid in shares (not diversified, liquid investments), this violates the fundamental right to ‘just and favourable remuneration’. The second major problem is that employee share schemes, which concentrate investment at firm level, break the cardinal rule of prudent portfolio theory: to diversify.^250^ Concentrating employees’ risks into shares would produce an ‘Enron’ economy.^251^ Successful cooperatives like John Lewis or Mondragon do not allow trading with shares: they are properly seen as employee partnerships. Their success derives from employees having the vote, not ownership in any correct sense. Moreover, with widespread automation there may be very few employees left in certain firms to own its shares. This is true of Alphabet (which controls Google) or most tech monopolies and networks. This is why the great architect of economic constitutionalism and economic rights in the 20th century, A.A. Berle, moved from being an advocate of share schemes,^252^ to seeking diversification by widely held pension funds.^253^ The goal is to democratise not just ownership but power.

### B. Universal Social Security

The universal right of everyone ‘as a member of society…to social security’ explicitly covers several elements of a welfare state, when people do not earn wages through a traditional employee-employer contract. In 1948, this highlighted ‘unemployment, sickness, disability, widowhood, old age’.^254^ A key function of social security is to ensure that the costs of unemployment are internalised by the state: if government has to pay social security bills it has a large incentive to boost aggregate demand to ensure fuller employment.^255^ This approach opposes the idea that mass unemployment or a minimum, basic income are acceptable. Instead, it complements the right to work for ‘just and favourable remuneration’.

By contrast, many social security systems leave people on income subsistence, and have major gaps, when the loss of a job, disability or old age is not their fault.^256^ Homeless people, and particularly people with learning difficulties, may struggle with bureaucratic registration requirements. If only available to women, child care pay may also entrench systemic gender-role segregation,^257^ and widen the gender pay gap. In the UK, women receive paid child care rights to a minimum level for 12 months, but although they may transfer a proportion to men the process is so complex that uptake is limited. In the USA, there is no right to paid family leave at all. Adult students often get nothing but debt. The solutions are to raise incomes to a fair level and fill the gaps, so that social security is truly universal: a default right to income unless someone is in employment or childhood education. It is also necessary to expand universal child care for all infants, as Eleanor Roosevelt’s original proposals made clear.^258^ The narrative of a ‘basic income’ is powerful for so many precisely because it could be part of universalising social security.

However, some basic income proposals are liable, without articulating details, to undermine other human rights, particularly plans that do not explain the tax source, or plans to replace (not complement) social security and labour rights. No universal income plan must act, as the Speenhamland disaster was shown to in part 3(A), to subsidise exploitative employers or landlords because this diminishes bargaining power and pay of all workers. It cannot be necessary for universality to make transfer payments to people who already pay income tax. To be taxed only to receive a sum back makes no sense. In countries like the USA where political democracy has been under sustained attack by the judiciary and corporate interests,^259^ handing power over incomes to a captured government threatens human development. Basic income programmes, like in Alaska, that sell commodities outside its system and are not funded by internal redistribution, are not models that can be generalised,^260^ because not every country has such resources. For this reason, the basic debate has begun to include universal basic services.^261^

The credible forms of basic income proposal (indeed, the *only* credible proposals) understand in detail behavioural psychology, tax and welfare law.^262^ In fact, introducing a ‘personal allowance’ in social security taxes (so that contributions are only made after a threshold) has the same economic effect as a basic income transfer for an employed person, but without the subsidy effect for employers.^263^ It also reduces bureaucracy. An essential principle, which upholds the universal right to ‘just and favourable remuneration’,^264^ is that tax is levied proportionately to the means to pay. For everyone not paying income tax, the legal right to income replacement, by auto-enrolment, would mean a tremendous advance in social prosperity. Most importantly, it would help ensure a universal fair income.

### C. Full Employment and Leisure

The ‘right to work’ and ‘holidays with pay’ are indivisible from the rights to ‘just and favourable’ pay, to share technology’s benefits and to social security. As society grows wealthier, there is a well established expectation, recognised in the European Social Charter 1961, for the ‘working week to be progressively reduced to the extent that the increase of productivity and other relevant factors permit’.^265^ Furthermore, the expansion of years in education, years in retirement, paid holidays and weekend days all increase people’s actual freedom. With more time off work people can pursue life’s true values: family, community, art, literature or sport. Recent empirical research suggests an optimal working week for people’s health could be up to 30, or as few as 8, hours.^266^ This is why the true meaning of ‘full employment in a free society’ is having the necessary hours of work and leisure ‘at fair wages’.^267^

The problem today is that unemployment and poverty are unacceptably high, while under-employment and inequality are growing. In many countries, working hours have increased and the retirement age has been raised. Like unfair wages, this distribution of work and leisure is a symptom of the benefits of science not being fairly shared, and substantially results from the anti-democratic nature of the economy. Far from a world, imagined by Keynes or Orwell, where the robots could work for us, all too often humans are turned into resources, and people become cogs in the machine. Worse than robots replacing people’s jobs, often it feels as if people themselves are being made into robots.^268^ Ultimately, this undermines the right to ‘the free and full development of [our] personality’.^269^

While collective bargaining and votes in the economy should lead the way in fair labour standards, minimum standards must be set by law, to foreclose unfair competition. Working time must be progressively reduced: the next logical steps are a three-day weekend, more holidays, expansion of child care, more years in education, and freezing or reducing the retirement age. While economic democratisation will limit the incentive of corporations to restrict investment in jobs and hoard capital, corporate profits also need to be taxed to encourage investment, not hoarding.^270^ Tax on hoarded property, which is not used in production, ensures assets are utilised and contributes to full employment. In addition, every central bank’s objective should be clarified to mean full employment, with real wage growth, and zero price inflation. Two or three per cent inflation, permanent wage attrition, and soaring inequality is not ‘price stability’ as most central bank laws require now.^271^ Again, as part 2(3) showed, the price tag of full employment is nearly zero, once business is confident that government will do ‘whatever it takes’. Once government commits to full employment, the idea of robots taking jobs will seem bizarre.

An alternative view, in a paper from the Obama White House, advocated policies of investing in artificial intelligence, educating and training for the ‘jobs of the future’ and to ‘aid workers in the transition’.^272^ This mirrors the EU employment strategy in seeing the need government investment as limited or negligible to reach the goal of full employment, and suggesting that private investment can be encouraged without any substantial use of fiscal and monetary policy.^273^ A ‘just transition’ to an automated world, in this vision, falls far short of the right to work and full employment. In the same category are proposals from Microsoft’s Bill Gates of ‘taxing the robots’ to pay for more human social services.^274^ This framing raised widespread concerns, until plans were shelved by the European Parliament, about robots acquiring legal personality. Such proposals are clearly misplaced. Like in corporate law, more legal personalities can be used as a way to evade legal responsibilities, such as compensation to tort victims,^275^ labour law or tax. The correct view is that robots are products^276^ and that robot owners (like Bill Gates) should pay their fair share of tax. It is fair that corporations like Microsoft and shareholders pay more in tax, because their profits bear some relation to work, more to luck and most to legal support.^277^ If everyone contributes proportionately to their means, there will be a truly ‘sharing economy’.

## 5. CONCLUSION

The promises of technology are astounding, and deliver humankind the capacity to live in a way that nobody could have once imagined. The industrial revolution of the 19th century brought people past subsistence agriculture. It became possible to live, not just from hand to mouth, bonded to lords and masters, but to win freedom from servitude through solidarity. The corporate revolution of the 20th century enabled mass production and social distribution of wealth, for human and democratic development across the globe. A third economic revolution has often been pronounced or predicted, but it will not only be one of technology. The next revolution will be social. It must be universal. Universal prosperity with democracy and social justice, on a living planet, is achievable not in centuries, but in years and decades. It did not begin with technology, but with education.^278^ Once people can see and understand the institutions that shape their lives, and vote in shaping them too, the robots will not automate your job away. There will be full employment, fair incomes and a thriving economy democracy.

## Footnotes

1 Oxfam, *An Economy for the 1%* (2016).

2 M. Zuckerberg, ‘Commencement Address at Harvard’ (25 March 2017).

3 D. Galeon, ‘Elon Musk: Automation Will Force Governments to Introduce Universal Basic Income’ (14 February 2017) Futurism.

4 M. Kubanzky and T. Williams, ‘Why We Invested: GiveDirectly’ (7 February 2017) omidyar.com.

5 V. Wadhwa, ‘Sorry, but the Jobless Future Isn’t a Luddite Fallacy’ (7 July 2015) *Washington Post*, cf *Closing Conversation with Jeff Bezos, Co-presented with SMU* (18 April 2018) George W. Bush, Presidential Centre, at 44:35.

6 Universal Declaration of Human Rights 1948 Art 27(1) and International Covenant on Economic, Social and Cultural Rights 1966 Art 15(1)(b).

7 For example, D.H. Autor, ‘Why Are There Still so Many Jobs? The History and Future of Workplace Automation’ (2015) 29(3) *Journal of Economic Perspectives* 3, 23.

8 See part 4(C) below and M. Friedman, ‘The Case for a Negative Income Tax: A View from the Right’ in J.H. Bunzel (ed.), *Issues in American Public Policy* (New York: Prentice-Hall, 1968) ch 2. G. Standing, *The Precariat: The New Dangerous Class* (London: Bloomsbury, 2016) 199–212.

9 C.B. Frey and M.A. Osborne, ‘The Future of Employment: How Susceptible Are Jobs to Computerisation?’ (2017) 114 *Technological Forecasting and Social Change* 254.

10 C.B. Frey, M. Osborne, With Contributions from Citi Research, ‘Technology at Work: The Future of Innovation and Employment’ (February 2015) Citi GPS. Osborne and Frey later distanced themselves from the original narrative that technology causes unemployment (13 April 2018) OxfordMartin Blog, ‘Our estimates have often been taken to imply an employment apocalypse. Yet that is not what we intended or suggested’. cf (2015) Citi GPS, 22, ‘There is concern that jobless economic recoveries have become the new normal…the concern over technological unemployment has **so far** proven to be exaggerated’. (*Emphasis added.*) ‘Agiletown: the relentless march of technology and London’s response’ (2014) Deloitte, Foreword, ‘Major advances in technology create a number of challenges…there will be a risk of avoidable unemployment’. Introduction, ‘We have welcomed the opportunity to work with Deloitte on this important research’. Frey has since produced ‘research’ with Uber saying its drivers are ‘happy’ and (in German) seems to oppose job security rights, e.g. (18 November 2016) faz.net, paraphrasing Frey, ‘If someone in Detroit or Cleveland loses their job, they must be flexible and mobile enough, to be able to work somewhere else in new branches’.

11 K. Grace et al., ‘When Will AI Exceed Human Performance? Evidence from AI Experts’ (30 May 2017) Working Paper.

12 F. Fukyama, *The End of History and the Last Man* (New York: Free Press, 1992).

13 cf *Back to the Future Part II* (1989).

14 For example, T. More, *Utopia* (1516, translated by P. Turner, London: Penguin) Book I. H. Sinzheimer, *Grundzüge des Arbeitsrechts* or *Foundations of Labour Law* (1920) ch 2, 22–7 (in German). A.A. Berle and G.C. Means, *The Modern Corporation and Private Property* (New York: Transaction, 1932) Part III.

15 UDHR 1948 Arts 22–25 and 27(1).

16 G. Orwell, *Animal Farm* (London: Secker and Warburg 1945) ch 5.

17 W. Beveridge, *Full Employment in a Free Society* (London: Allen and Unwin, 1944), see below at part 4(A).

18 Note ‘Cognition’ means the ‘process of acquiring knowledge and understanding through thought, experience, and the senses’.

19 For example, ‘Octuplets’ Mom on Welfare, Spokesman Confirms’ (10 February 2009) *Fox News*. A. Crick and K. Cain, ‘Benefit Mum Rap: “Queen of Benefits”’ (21 February 2017) *The Sun*. S. Markson, ‘Taxpayer Works for 14 Years to Pay Welfare Bill of Just ONE Dole Bludger’ (22 June 2017) *Daily Telegraph*. Each ‘news’ outlet is controlled by Victorian mogul, Rupert Murdoch.

20 cf A. Budd, *Pandora’s Box, Episode 3: The League of Gentlemen* (Thursday, 18 June 1992) BBC2 at 42:55.

21 UDHR 1948 Arts 22–23.

22 M. Friedman, ‘The Role of Monetary Policy’ (1968) 58(1) *AER* 1, 10.

23 Friedman (1968) 58(1) *AER* 1, 9. Note, inflation typically diminishes real wages, but enriches owners and sellers of assets.

24 Friedman (1968) 58(1) *AER* 1, 11.

25 Friedman (1968) 58(1) *AER* 1, 9.

26 A.W. Phillips, ‘The Relation between Unemployment and the Rate of Change of Money Wage Rates in the United Kingdom 1861–1957’ (1958) 25 *Economica* 283.

27 Unequal bargaining power is consequent on three main factors: (1) inequality in resources or wealth: A Smith, *The Wealth of Nations* (1776) Book I, ch 8, §12, (2) collective organisation: JS Mill, *Principles of Political Economy* (1848) Book V, ch XI, §12, and (3) asymmetry of information: WS Jevons, *Theory of Political Economy*, 3rd edn (1888) ch 4, §74.

28 M. Friedman, ‘The Methodology of Positive Economics’ in *Essays in Positive Economics* (Chicago: University of Chicago Press, 1953) ch 1, 4–7.

29 Note D. Hume, *Essays Moral, Political, Literary* (1777) Part II, Essay III, *Of Money*.

30 F.A. Hayek, ‘Full Employment, Planning and Inflation’ (1950) 4(6) *Institute of Public Affairs Review* 174.

31 F.A. Hayek, *The Road to Serfdom* (London: Routledge, 1944).

32 Hayek (1950) 4(6) *IPAR* 174, 181.

33 For example, M.L. Wachter, ‘The Nature of the Unemployment Problem’ (1978) 21(2) *Challenge* 32, estimating 5.5%.

34 BP, *Statistical Review of World Energy* (June 2017) 20.

35 The only better 16 years in recent UK history was 1991–2006, at 2.76%. For 1968–72, around the Industrial Relations Act 1971, inflation ranged from 6.4 to 9.4%. P. Davies and M. Freedland, *Labour Legislation and Public Policy* (OUP, 1992) chs 6 and 7, 365.

36 M. Friedman, ‘Inflation and Unemployment’ (1977) 85 *Journal of Political Economy* 451, 458.

37 Friedman (1977) 85 *JPE* 451, 458.

38 Friedman (1977) 85 *JPE* 451, 462–3.

39 Friedman (1977) 85 *JPE* 451, 464–5.

40 See shortly after, J.A. Tatom, ‘What Ever Happened to the Phillips Curve?’ (1981) Federal Reserve Bank of St Louis Working Paper 1981-008A, 3.

41 D.T. Coe, ‘Nominal Wages. The NAIRU and Wage Flexibility’ (1985) 5 *OECD Economic Studies* 87, 88.

42 S.P. Hargreaves Heap, ‘Choosing the Wrong “Natural” Rate: Accelerating Inflation or Decelerating Employment and Growth?’ (1980) 90(359) *Economic Journal* 611.

43 D.J. Burningham ‘Economics for Technical Students’ (1960) 2(1) Education + Training 11, 12–13. ES Phelps, *Inflation Policy and Unemployment Theory: The Cost-Benefit Approach to Monetary Planning* (1972) xxiii (applying the analogy, but sadly accepting a ‘natural’ rate) and Hargreaves Heap (1980) 90(359) *Economic Journal* 611.

44 K. Clark and L. Summers, ‘Labour Force Participation: Timing and Persistence’ (1982) 49(5) *Review of Economic Studies* 825. O.J. Blanchard and L.H. Summers, ‘Hysteresis and the European Unemployment Problem’ (1986) 1 *NBER Macroeconomics Annual* 16. Furthermore, ‘Hysterisis in Unemployment’ (1987) 31 *European Economic Review* 288. ‘Beyond the Natural Rate Hypothesis’ (1988) 78(2) *AER* 182, 186.

45 For example, R.M. Solow, ‘On Theories of Unemployment’ (1980) 70(1) *AER* 1, on ‘lesser mortals’.

46 R. Layard et al., ‘Special Issue on Unemployment’ (1982) 49(5) *RES* 675.

47 D. Grubb, R. Jackman and R. Layard, ‘Causes of the Current Stagflation’ (1982) 49(5) *RES* 707.

48 M. Andrews and S. Nickell, ‘Unemployment in the United Kingdom Since the War’ (1982) 49(5) *RES* 731, 751.

49 Andrews and Nickell (1982) 49(5) *RES* 731, 741. Furthermore, D.T. Mortensen and C.A. Pissarides, ‘Job Creation and Job Destruction in the Theory of Unemployment’ (1994) 61(3) *Review of Economic Studies* 397.

50 S. Bentolila and G. Bertola, ‘Firing Costs and Labour Demand: How Bad Is Eurosclerosis?’ (1990) 57 *Review of Economic Studies* 381, 395–6 and 400, admitting: ‘We do not have data on the actual rise of [firing costs] in the seventies; we set the increase to 33% when solving the model’.

51 Bentolila and Bertola (1990) 57 *Review of Economic Studies* 381, 394.

52 OECD, *Jobs Study, Evidence and Explanations* (1994). cf D. Ashiagbor, *The European Employment Strategy: Labour Market Regulation and New Governance* (2005) ch 2, 38–48.

53 P. Krugman, ‘Past and Prospective Causes of High Unemployment’ (1994) 79(4) *Economic Review - Federal Reserve Bank of Kansas City* 23, 50, 57 and 74.

54 Krugman (1994) 79(4) *Economic Review* 23, 74–76.

55 UDHR 1948 Arts 22–23.

56 Bank of England Act 1998 s 11. TFEU 2007 Art 127. M. Roth, ‘Employment as a Goal of Monetary Policy of the European Central Bank’ (2015) ssrn.com.

57 J.L. Yellen, ‘Monetary Policy: Goals and Strategy’ (13 March 1996) NABE Policy Seminar, Washington, DC.

58 D. Staiger, J.H. Stock and M.W. Watson, ‘The NAIRU, Unemployment and Monetary Policy’ (1997) 11(1) *Journal of Economic Perspectives* 33, the CBO estimated 5.8% and Council of Economic Advisers 5.7%. On the actual rate, see below.

59 A. Paz-Fuchs, *Welfare to Work: Conditional Rights in Social Policy* (Oxford: OUP, 2008) ch 3.

60 S. Nickell, ‘Unemployment and Labor Market Rigidities: Europe versus North America’ (1997) 11 *Journal of Economic Perspectives* 55, 66.

61 R. Layard, ‘Getting people back to work’ (1998) Autumn, CentrePiece 24, 27.

62 For example, O. Blanchard and J. Wolfers, ‘The Role of Shocks and Institutions in the Rise of European Unemployment: The Aggregate Evidence’ (2000) 110 *Economic Journal* 1. D.H. Autor, J.J. Donohue III and S.J. Schwab, ‘The Costs of Wrongful-Discharge Laws’ (2006) 88(2) RES 211. L.H. Summers, ‘Unemployment’ in *The Concise Encyclopedia of Economics*, 2nd edn (2007) econlib.org.

63 Z. Adams, L. Bishop, S. Deakin, C. Fenwick, S. Martinsson and G. Rusconi, ‘The Economic Significance of Laws Relating to Employment Protection and Different Forms of Employment: Analysis of a Panel of 117 Countries, 1990–2013’ (2019) 158(1) *International Labour Review* 1. This is based on the CBR, Labour Regulation Index (2016).

64 V.V. Acharya, R.P. Baghai and K.V. Subramanian, ‘Labor Laws and Innovation’ (2013) 56(4) *Journal of Law and Economics* 997.

65 cf L. Ball and N.G. Mankiw, ‘The NAIRU in Theory and Practice’ (2002) 16(4) *Journal of Economic Perspectives* 115 and J.K. Galbraith, ‘Time to Ditch the NAIRU’ (1997) 11(1) JEP 93.

66 J.P. Morgan, *Paychecks, Paydays, and the Online Platform Economy* (2016) 55% of 6m customers had 30% volatility in monthly wages.

67 D. Ashiagbor, *The European Employment Strategy: Labour Market Regulation and New Governance* (Oxford: OUP, 2005), ch 2, 65–6.

68 Y. Li, ‘The Fed Chairman Says the Relationship between Inflation and Unemployment Is Gone’ (11 July 2019) *CNBC*.

69 J.M. Keynes, *The Economic Possibilities of Our Grandchildren* (1930).

70 See also E Skidelsky and R Skidelsky, *How Much is Enough?: Money and the Good Life* (London: Penguin, 2013) Preface and ch 1.

71 J. Schumpeter, *Capitalism, Socialism and Democracy* (London: Harper, 1942) chs VII and VIII.

72 Schumpeter (1942) ch VIII, 90.

73 G. Orwell, *Animal Farm* (London: Secker and Warburg, 1945) ch 5.

74 J. Rifkin, *The End of Work: The Decline of the Global Labor Force and the Dawn of the Post-Market Era* (New York: GP Putnam’s Sons. 1995).

75 Rifkin (1995) ch 5, 71–3, mechanical harvester use from 6% to 78% in 15 years.

76 M.L. King, *Speech to the Fourth Constitutional Convention AFL-CIO, Miami, Florida* (11 December 1961) says the opposite.

77 Rifkin (1995) ch 10, 149.

78 Rifkin (1995) ch 7, 257–65. See further B Dorini, Review (1996) 9(1) *Harvard Journal of Law & Technology* 231.

79 E. Brynjolfsson and A. McAffee, *Race Against the Machine: How the Digital Revolution is Accelerating Innovation, Driving Productivity, and Irreversibly Transforming Employment and the Economy* (Digital Frontier Press, 2011) ch 1.

80 Brynjolfsson and McAffee (2011) ch 5. They also call Friedrich von Hayek ‘the greatest theorist of markets’. This is debatable.

81 E. Brynjolfsson and A. McAfee, *The Second Machine Age: Work, Progress, and Prosperity in a Time of Brilliant Technologies* (2014) ch 14.

82 See campaignmoney.org, on Brynjolfsson contributing to ActBlue and Bernie 2016 and McAfee to the Hillary Victory Fund.

83 C.L. Estlund, ‘The Ossification of American Labour Law’ (2002) 102(6) *Columbia Law Review* 1527.

84 See E. McGaughey, ‘Fascism-lite in America (or the social ideal of Donald Trump)’ (2018) 7(2) *BJALS* 291.

85 cf E. McGaughey, ‘A Human Is Not a Resource’ (2020) 31(2) *King’s Law Journal* 1, and ‘Votes at Work in Britain: Shareholder Monopolisation and the “Single Channel”’ (2018) 47(1) *ILJ* 76.

86 Worker Adjustment and Retraining Notification Act of 1988 (29 USC §§ 2101–9).

87 Employment Rights Act 1996 ss 86 (notice), 94 (right to a fair dismissal) and 135 (redundancy pay).

88 Note, the rise in UK unemployment from 2010 to 2012, against the trend down in the USA and Germany was created by the Cameron-Clegg coalition choice to inflict an ‘age of austerity’ on the UK. It led to the longest wage falls in modern history, eg ‘Drop in Real Wages Longest for 50 Years, Says ONS’ (31 January 2014) *BBC News*.

89 E. McGaughey, ‘How Do We Stop an Unemployment Pandemic?’ (19 May 2020) *Guardian*.

90 Work Constitution Act 1972 (*Betriebsverfassungsgesetz*) §§111–113. Dismissal Protection Act 1965 (*Kündigungschutzgesetz*) §1.

91 For example, ‘Buying Itself Out of a Recession’ (4 November 2009) Spiegel.

92 R. Krause and J.W. Kühn, ‘COVID-19 and Labour Law: Germany’ (2020) 13(1S) *Italian Labour Law eJournal* 1, 2.

93 W. Beveridge, *Full Employment in a Free Society* (London: Allen and Unwin, 1944) 18.

94
*Braganza v BP Shipping Ltd* [2015] UKSC 17, [18].

95 Z. Adams, L. Bishop, S. Deakin, C. Fenwick, S. Martinsson and G. Rusconi, ‘The Economic Significance of Laws Relating to Employment Protection and Different Forms of Employment: Analysis of a Panel of 117 Countries, 1990–2013’ (2019) 158(1) *International Labour Review* 1. This is based on the CBR, *Labour Regulation Index* (2016).

96 C.B. Frey and M.A. Osborne, ‘The Future of Employment: How Susceptible Are Jobs to Computerisation?’ (2017) 114 *Technological Forecasting and Social Change* 254.

97 Frey and Osborne (2017) 114 TFSC 254, 257.

98 Frey and Osborne (2017) 114 TFSC 254, 268.

99 Frey and Osborne (2017) 114 TFSC 254, 263.

100 C.B. Frey, M. Osborne, With Contributions from Citi Research, ‘Technology at Work: The Future of Innovation and Employment’ (February 2015) Citi GPS.

101 McKinsey Global Institute, *A Future That Works: Automation, Employment, and Productivity* (2017).

102 PwC, *UK Economic Outlook March 2017* (2017) ch 4.

103 Deloitte, *Citizens, Government and Business: The State of the State 2017–18* (2017) 10.

104 K. Grace et al., ‘When Will AI Exceed Human Performance? Evidence from AI Experts’ (30 May 2017) Working Paper.

105 For example, D.H. Autor, ‘Why Are There Still So Many Jobs? The History and Future of Workplace Automation’ (2015) 29(3) *Journal of Economic Perspectives* 3.

106 Executive Office of the President, *Artificial Intelligence, Automation and the Economy* (December 2016) 2 and 13–19.

107 Smith (1776) Book II, ch 1.

108 A.A. Berle, ‘Property, Production and Revolution’ (1965) 65 *Columbia Law Review* 1. H. Sinzheimer, *Grundzüge des Arbeitsrechts* (1920) ch 2, 22–27.

109 J.M. Keynes, *The General Theory of Employment, Interest and Money* (London: Macmillan, 1936) ch 12 and *The Economic Possibilities of our Grandchildren* (1930).

110 For example, C. Pigou, *The Theory of Unemployment* (Taylor & Francis, 1933) or J.S. Mill, *Principles of Political Economy* (London: John W. Parker, 1848) Book II, ch XII, §1.

111 Keynes (1936) chs 2 and 3, arguing workers cannot bargain for higher *real* wages because this also depended on price levels.

112 Keynes (1936) ch 9.

113 Keynes (1936) ch 12, V, 149.

114 Keynes (1936) ch 12, III, 145.

115 Keynes (1936) ch 22.

116 Keynes (1936) ch 15.

117 Keynes (1936) ch 12, VI.

118
*Campaign Address on Progressive Government at the Commonwealth Club in San Francisco, California* (1932).

119 G.C. Means, ‘The Separation of Ownership and Control in American Industry’ (1931) 46(1) *QJE* 68. This followed *State Board of Control v Buckstegge*, 158 Pac 837, 842 (1916), *Busser v Snyder*, 282 Pa 440 (1925) and *Railroad Retirement Board v Alton Railroad Co* 295 US 330 (1935).

120 Berle and Means (1932) Part III. cf M. Friedman and A.J. Schwarz, *A Monetary History of the United States, 1867–1960* (Princeton, NJ: Princeton UP. 1963).

121 See *Steward Machine Company v Davis* 301 US 548 (1937) following shortly after *West Coast Hotel Co v Parrish* 300 US 379 (1937).

122 Social Security Act of 1935, 42 USC §402.

123 For example, S Labaton, ‘Bush S.E.C. Pick Is Seen as Friend to Corporations’ (3 June 2005) NY Times. In the UK, see the notorious Opinion of Robin Potts QC to the International Swaps and Derivatives Association, Inc (24 June 1997).

124 E. Warren, ‘Product Safety Regulation as a Model for Financial Services Regulation’ (2008) 43(2) *Journal of Consumer Affairs* 452, cf Consumer Credit Act 1974 and Unfair Terms Directive 93/13/EEC Arts 3–6.

125 M.S. Eccles, *Beckoning Frontiers: Public and Personal Recollections* (New York: Alfred A. Knopf, 1951) 75–6.

126 Eccles (1951) 77.

127 Roosevelt (1932) ‘princes of property…must fearlessly and competently assume the responsibility which goes with the power’. German Constitution 1949 §14(2) ‘Eigentum verpflichtet’.

128 White Paper, *Employment Policy* (May 1944) Cmd 6527, [43]-[45] and [57]-[63], also summarised by M. Kalecki (1944) 6(8) *Oxford Bulletin of Economics and Statistics* 131.

129 Disabled Persons (Employment) Act 1944 s 9.

130 Distribution of Industry Act 1945 and 1950.

131
*Distribution of Industry* (1948) Cmd 7540, 26.

132 Hansard HC Debs (21 March 1945) vol 409, col 859. John Wardlaw-Milne MP was never very popular in his party.

133 S. Webb, *How the Government Can Prevent Unemployment* (1912) 6–9. A. Briggs, *Social Thought and Social Action: A Study of the Work of Seebohm Rowntree 1871– 1954* (London: Longmans, 1961).

134 Webb (1912) 12. See part 3(1) below.

135 M. Kalecki, ‘Political aspects of full employment’ (1943) 14(4) *Political Quarterly* 322.

136 C. Shapiro and J.E. Stiglitz, ‘Equilibrium Unemployment as a Worker Discipline Device’ (1984) 74(3) *AER* 433.

137 Another, tremendous problem was a sexist assumption of men being entitled to jobs.

138 AA Berle, ‘A New Look at Management Responsibility’ (1962) 2 *Human Resource Management* 3.

139 R.C.O. Matthews, ‘Why Has Britain Had Full Employment since the War?’ (1968) 78(311) *Economic Journal* 555.

140 See also B. Hansen, W.W. Snyder and OECD, ‘Fiscal Policy in Seven Countries 1955–1965: Belgium, France, Germany, Italy, Sweden, United Kingdom, United States’ (1969) 0501/QL 200 H249.

141 ONS, *Coronavirus Job Retention Scheme Statistics* (December 2020) Table 1, on 15 May, 8,815,700 furloughed, Table 8, £43.3 bn.

142 Kalecki (1943) 14(4) *Political Quarterly* 322, 330.

143 T. Opler, L. Pinkowitz, R. Stulz and R. Williamson, ‘The Determinants and Implications of Corporate Cash Holdings’ (1999) 52 *Journal of Financial Economics* 3, 18 (showing decline in corporate hoarding from the 1950s till the 1990s).

144 X. Gao, ‘Corporate Cash Hoarding: The Role of Just-in-Time Adoption’ (2018) *Management Science* (forthcoming), 6.

145 P. Seitz, ‘Apple Has Largest Cash Stockpile, Twice That of Microsoft’s’ (20 May 2016) *Investor’s Business Daily*.

146 In November 2017, the number of unemployed was 6.6m. A $10 an hour job, for 38 hours a week, for 50 weeks in a year means $22,914 in pre-tax ($19,090.90 post-tax) annual income. That means 26.4 million jobs could be created with $504 billion. This calculation vastly *overestimates* the expense of full employment: the multiplier effect of such investment would be colossal.

147 cf Berle and Means (1932) Book I, ch 1.

148 More, *Utopia*, Book I, 25. More’s own views were probably those of Raphael Nonsenso.

149 More (1516) Book I, 27.

150 Vagabond Act 1495. R. Pashley, *Pauperism and Poor Laws* (Cambridge: Cambridge UP, 2011) ch 5.

151 See further E.H. Shagan, ‘Protector Somerset and the 1549 Rebellions: New Sources and New Perspectives’ (1999) 114(455) *English Historical Review* 34. D. MacCulloch, ‘Kett’s Rebellion in Context’ (1979) 84 *Past & Present* 36.

152 See P.J. Federico, ‘Origin and Early History of Patents’ (1929) 11 *Journal of the Patent Office Society* 292, 298 and M. Grass and A. Grass, *Stockings for a Queen: the life of the Rev. William Lee, the Elizabethan Inventor* (1967) 122.

153 S.D. Chapman, ‘Enterprise and Innovation in the British Hosiery Industry, 1750–1850’ (1974) 5(1) *Textile History* 14, 24.

154 See S. Webb and B. Webb, *The History of Trade Unionism* (1920) ch II, 88 ff.

155 Lord Byron, Hansard HL Debs (27 February 1812) vol 21, col 966. See also C. Brontë, *Shirley* (1849).

156 Hansard HL Debs (27 February 1812) vol 21, col 969, ‘the sword is the worst argument…so should it be the last: in this instance it has been the first, but…as yet, only in the scabbard. The present measure will…pluck it from the sheath…’.

157 See G. Clark, ‘The Long March of History: Farm Wages, Population and Economic Growth, England 1209–1869’ (2007) 60(1) *Economic History Review* 97. N.F.R. Crafts and T.C. Mills, ‘Trends in Real Wages in Britain, 1750–1913’ (1994) 31(2) *Explorations in Economic History* 176. Wage declines did recur after TUA 1871, but were less prolonged and severe, until 2010. See TUC, ‘UK Workers Suffering the Most Severe Squeeze in Real Earnings since Victorian Times’ (12 October 2014).

158 See S. Deakin and F. Wilkinson, *The Law of the Labour Market: Industrialization, Employment and Legal Evolution* (Oxford: OUP, 2005) 63–7.

159
*R v Journeymen-Taylors of Cambridge* (1721) 88 ER 9.

160 Smith (1776) Book I, ch 8, §12.

161 K. Polayni, *The Great Transformation* (New York: Farrar & Rinehart. 1944) chs 7–9, especially 82.

162 Deakin and Wilkinson, *The Law of the Labour Market: Industrialization, Employment and Legal Evolution* 125–7.

163 cf T.R. Malthus, *An Essay on the Principle of Population* (London: J. Johnson, 1798).

164 The Great Reform Act 1832 extended the vote in Parliament, yet still to a tiny percentage of the male population.

165 cf R. Bregman, ‘No, Wealth Isn’t Created at the Top. It Is Merely Devoured There’ (30 March 2017) *Guardian*.

166 E. Hobsbawm and G. Rudé, *Captain Swing* (London: Phoenix, 1969) 50–1.

167
*R v Lovelass* (1834) 172 ER 1380.

168 S. Webb and B. Webb, *The History of Trade Unionism* (London: Longmans, Green and Co, 1920) ch III, 144–53.

169 H. Feis, ‘The Industrial Situation in Great Britain: From the Armistice to the Beginning of 1921’ (1921) 11(2) *American Economic Review* 265–6, predicted this.

170 T. Wilson, ‘Notes on Labour Unrest’ (1919) 29(115) *Economic Journal* 367, 372.

171 S. Ward, *Unemployment and the State in Britain: The Means Test and Protest in 1930s* (Manchester: Manchester UP, 2013) 34–6, and *The Times* (2 January 1919) 3. cf Deakin and Wilkinson (2005) 160–3.

172 O.T. Mallery, ‘Preventing Periods of Unemployment by Expanding Public Works’ (1921) *American Labor Legislation Review* 47.

173 See N. Ferguson, ‘Germany after the First World War by Richard Bessel’ (1995) 110(439) *English Historical Review* 1224.

174 R. Bessel, *Germany After the First World War* (Oxford: Clarendon, 1993) ch 5.

175 Emergency Tariff of 1921 and the Fordney–McCumber Tariff of 1922.

176 J.M. Keynes, *The Economic Consequences of the Peace* (New York: Harcourt, Brace and Howe, 1920).

177 A.R. Seipp, *The Ordeal of Peace: Demobilization and the Urban Experience in Britain and Germany, 1917–1921*, 2nd edn (London: Routledge, 2016) ch 7.

178 E. McGaughey, ‘The Codetermination Bargains: The History of German Corporate and Labor Law’ (2016) 23(1) *Columbia Journal of European Law* 135, 163–4.

179 G. Miller, ‘The Economic Policy of British Labor’ (1946) 6(2) *Antioch Review* 179, 183.

180 White Paper, *Employment Policy* (1944) Cmd 6527, 7, §11.

181 (1944) Cmd 6527, 7–8, §14, and Reinstatement in Civil Employment Act 1944.

182 Servicemen’s Readjustment Act of 1944 and Veterans’ Preference Act of 1944.

183 Employment Act of 1946, 15 USC §1021. See K.V.W. Stone, ‘A Right to Work in the United States’ in V Mantouvalou (ed) *The Right to Work* (Oxford: Hart, 2015) ch 15.

184 G. Marshall, *The Marshall Plan Speech* (5 June 1947) Harvard.

185 UDHR 1948 Art 23(1).

186 T. Wheelwright, *Oil and World Politics* (Left Book Club, 1991) ch 1.

187 H.B. Brown, ‘The Status of the Automobile’ (1908) 17(4) *Yale Law Journal* 223, 227.

188 Brown (1908) 17(4) *Yale Law Journal* 223, 225–7.

189 E.R. Kilby, ‘The Demographics of the U.S. Equine Population’ in *The State of America’s Animals IV* (Washington, DC: Humane Society Press, 2007) ch 10, 176.

190 E. Albin, ‘Labour Law in a Service World’ (2010) 73(6) *MLR* 959.

191 World Health Organization, *Global Status Report on Road Safety 2015* (2015) Table A2.

192 cf Brodsky (2016) 31(2) *Berkeley Tech LJ* 851, 862, a ‘car is a product’, 866, accidents could be reduced by 80%.

193 J. Langan-Fox et al. (ed), *Handbook of Stress in the Occupations* (Cheltenham: Edward Elgar, 2011) ch 21.

194 H. Rosling, *The Magic Washing Machine* (2010) youtube.com.

195 C.E. Beecher and H. Beecher Stowe, *American Woman’s Home: Or, Principles of Domestic Science* (1842) ch XXV.

196 K. French-Fuller, ‘Gendered Invisibility, Respectable Cleanliness: The Impact of the Washing Machine on Daily Living in Post-1950 Santiago, Chile’ (2006) 18(4) *Journal of Women’s History* 79.

197 J.C. Robinson, ‘Of Women and Washing Machines’ (1985) 101 *China Quarterly* 32.

198 L. Hannah, *Inventing Retirement: The development of Occupational Pensions in Britain* (Cambridge: CUP, 1986) 13.

199 Chart from Google’s ngram, showing the incidence of words in books.

200 For example, ILO Hours of Work (Industry) Convention 1919 (c 1) and then FT Vyver, ‘Regulation of Wages and Hours Prior to 1938’ (1939) 6(3) Law and Contemporary Problems 323.

201 H. Rosling, *The Magic Washing Machine* (2010) youtube.com.

202 On eliminating the gender pay gap, see E. McGaughey (2017) 46(1) *ILJ* 169, 179–80.

203 S. Strasser, *Never Done: A History of American Housework* (Henry Holt and Co. 1982) ch 5, on piped water in Philadelphia increasing consumption x4.

204 One might also point to public education and health, which are the basis of ideas for all technology.

205 D. Spencer and G. Slater, ‘No Automation Please, We’re British: Technology and the Prospects for Work’ (2020) 13 *Cambridge Journal of Regions, Economy and Society* 117.

206
*Terminator 2: Judgment Day* (1991) starring L Hamilton and A Schwarzenegger.

207 J. Bessen, ‘How Computer Automation Affects Occupations: Technology, Jobs, and Skills’ (2016) *Boston University, Law & Economics* WP No. 15–49.

208 N.G. Leigh, B. Kraft and H. Lee, ‘Robots, Skill Demand and Manufacturing in US Regional Labour Markets’ (2020) 13 *Cambridge Journal of Regions, Economy and Society* 77.

209 C. Markou and S. Deakin, ‘Ex Machina Lex: Exploring the Limits of Legal Computability’ in *Is Law Computable? Critical Perspectives on Law and Artificial Intelligence* (London: Bloomsbury, 2020) ch 2.

210 Y. LeCun, Y. Bengio and G. Hinton, ‘Deep Learning’ (2015) 521 *Nature* 436.

211 Markou and Deakin (2020) ch 2.

212 The answer, of course, is ‘to get a byte to eat’.

213 But cf T. Hobbes, *De Corpore* (1655) ‘By reasoning I understand computation’. Explained in S. Deakin and C. Markou, ‘From Rule of Law to Legal Singularity’ in *Is Law Computable? Critical Perspectives on Law and Artificial Intelligence* (London: Bloomsbury, 2020) ch 1.

214 n.b. ‘Intelligence’ means ‘the ability to acquire and apply knowledge and skills’. From the Oxford Dictionary of English.

215 On the meaning of ‘cognition’, see n 18, above.

216 See Q. Skinner, *Visions of Politics* (Cambridge: CUP, 2002) vol 1, ch 2, ‘The Practice of History and the Cult of Fact’.

217 cf Aristotle, *Nicomachean Ethics*, Book V, Pts 3–4, who argued ‘corrective’ justice is arithmetic; ‘distributive’ justice is geometric.

218 See D. Silver, ‘Mastering the Game of Go Without Human Knowledge’ (19 October 2017) 550 *Nature* 354 and cf G. Kasparov and M. Greengard, *Deep Thinking: Where Machine Intelligence Ends and Human Creativity Begins* (London: John Murray, 2017).

219 See R. Descartes, *Discourse on the Method* (1637) Part 4. It is likely his famous ‘eureka’ moment was simmering for some time.

220 L. Wittgenstein, *Tractatus Logico-Philosophicus* (1921).

221 L. Wittgenstein, *Philosophical Investigations* (1953) §43. cf G. Kitching, *Wittgenstein and Society: Essays in Conceptual Puzzlement* (Aldershot: Ashgate, 2003).

222 See also E. Durkheim, *Division of Labour in Society* (1893) 165–6.

223 See also International Covenant on Economic, Social and Cultural Rights 1966 Art 15.

224 UNESCO, *Venice Statement on the Right to Enjoy the Benefits of Scientific Progress and its Applications* (2009) §§12(d) and 16.

225 L. Shaver, ‘The Right to Science and Culture’ [2010] *Wisconsin Law Review* 121, 143 and 138–9.

226 UNDP, *Human Development Indices and Indicators: 2018 Statistical Update* (2018) 30.

227 R.B. Freeman, ‘Who Owns the Robots Rules the World’ (May 2015) IZA World of Labor.

228 For example, A. Yang (30 April 2018) Twitter, and T. Riley, ‘What Andrew Yang’s Universal Basic Income Would Actually Look Like’ (23 April 2019) Mother Jones.

229 Executive Office of the President, *Artificial Intelligence, Automation and the Economy* (December 2016) 28 ff.

230 UDHR 1948 Art 23(4).

231 A. Smith, *The Wealth of Nations* (1776) Book I, ch 8, §12. NLRA 1935 §1.

232 E.A. Posner, F.M.S. Morton and E.G. Weyl, ‘A Proposal to Limit the Anti-Competitive Power of Institutional Investors’ (2017) 81 *Antitrust LJ* 1, 2.

233 S. Krouse, D. Benoit and T. McGinty, ‘Meet the New Corporate Power Brokers: Passive Investors’ (October 24, 2016) *Wall Street Journal*.

234 S. Hirst, ‘Social Responsibility Resolutions’ (2018) 43 *Journal of Corporation Law* 217, 224–27.

235 cf Smith (1776) Book V, ch 1, §107. L.D. Brandeis, *Other People’s Money and How the Bankers Use It* (1914).

236 T. Piketty, *Capital in the Twenty-First Century* (Cambridge, MA: Belknap, 2014) ch 2, 25–26 and 351.

237 McGaughey (2017) 46(1) *ILJ* 169.

238 E. McGaughey, ‘The Codetermination Bargains: The History of German Corporate and Labor Law’ (2016) 23(1) *Columbia Journal of European Law* 135, on how codetermination came from collective agreements, later codified by law.

239 E. McGaughey, ‘Votes at Work in Britain: Shareholder Monopolisation and the “Single channel”’ (2018) 47(1) *ILJ* 76, explaining the evolution of pension codetermination in the UK. E. McGaughey, ‘Democracy in America at Work: The History of Labor’s Vote in Corporate Governance’ (2019) 42 *Seattle University Law Review* 697, boardroom and pension democracy in the USA.

240 E. McGaughey, *Principles of Enterprise Law* (Cambridge: CUP, 2021) Forthcoming.

241 For example, Adams et al, ‘The Economic Significance of Laws Relating to Employment Protection’.

242 cf Reward Work Act of 2018 (s 2605), the Accountable Capitalism Act of 2018 (s 3348) and the ‘Shareholder Democracy’ section of the ‘Corporate Accountability and Democracy’ plan of Bernie Sanders. For the UK, see K.D. Ewing et al, *Rolling out the Manifesto for Labour Law* (IER, 2018) ch 5.

243 Following the ‘People’s Initiative against Rip-off Salaries’ (2013).

244 For example, Education Reform Act 1988 s 124A, and Sch 7A, para 3, requiring up to two students on governing bodies. cf H Morrison, *Socialisation and Transport* (1933) ch XI, 197, rejecting that passengers or workers should have voting rights in public enterprise. Unfortunately, this set the pattern for much UK nationalised industry. Today, see S. Deakin, ‘The Corporation as Commons’ (2012) 37(2) *Queen’s Law Journal* 339.

245 For example, in the Model Articles for Public Companies, Sched 3, a new para 34(2) could state: ‘The company’s workers shall be registered as members and entitled to 30 per cent of the total votes in the general meeting. Each person shall have one vote’.

246 Delaware General Corporation Law of 1969 §141(h). UK Model Articles, Sch 3, para 23(2).

247 E. McGaughey, ‘Does Corporate Governance Exclude the Ultimate Investor?’ (2016) 16(1) *JCLS* 221, 237–8.

248 See E. McGaughey and M. Lawrence, ‘The Green Recovery Act 2020’ (July 2020) CommonWealth.

249 R.B. Freeman, ‘Who Owns the Robots Rules the World’ (May 2015) IZA World of Labor.

250 McGaughey (2018) 47(1) ILJ 76 and see the Trustee Act 2000 s 4(3)(b) on the duty to diversify.

251 P.J. Purcell, ‘The Enron Bankruptcy and Employer Stock in Retirement Plans’ (11 March 2002) CRS Report for Congress.

252 A.A. Berle, ‘How Labor Could Control’ (1921) 28 *New Republic* 37.

253 A.A. Berle, ‘Property, Production and Revolution’ (1965) 65 *Columbia Law Review* 1, 17.

254 UDHR 1948 Art 25.

255 cf T. Paine, *Agrarian Justice* (1797).

256 Contrast Germany’s Social Code, the US Social Security Act of 1935, and hybrids like in Sweden.

257 A.C.L. Davies, *Perspectives on Labour Law* (Oxford: OUP, 2004) 111

258
*American Women: Report of the President’s Commission on the Status of Women* (1963) 22. L. Crehan, *Cleverlands* (London: Unbound 2018) chs 2–5.

259 E. McGaughey, ‘Fascism-Lite in America (or the Social Ideal of Donald Trump)’ (2018) 7 BJALS 291.

260 cf J. Hickel, *The Divide* (London: Windmill, 2017) ch 9.

261 J. Portes, H. Moore et al, *Social Prosperity for the Future: A Proposal for Universal Basic Services* (Institute for Global Prosperity, 2017).

262 G. Standing, *Basic Income: And How We Can Make It Happen* (Pelican, 2017) ch 7.

263 cf S. Deakin and F. Wilkinson, ‘Labour Law, Social Security and Economic Inequality’ (1991) 15 *Cambridge Journal of Economics* 125, 144–5.

264 UDHR 1948 Art 23(3).

265 European Social Charter 1961 Art 2(1).

266 D. Kamerade, S. Wang, B. Burchell, S.U. Balderson and A. Coutts, ‘A Shorter Working Week for Everyone: How Much Paid Work Is Needed for Mental Health and Well-Being?’ (2019) *Social Science & Medicine*.

267 Beveridge, *Full Employment in a Free Society* (1944) 18.

268 V. Pietrogiovanni, ‘The Robotisation of Work or the Robotisation of Workers?: The Impact of AI and Digitisation on Employer’s Powers and Employee’s Obligations’ (2017) presented at Labour 2030 – Rethinking the Future of Work.

269 UDHR 1948 Art 29.

270 M.R. Cohen, ‘Property and Sovereignty’ (1927) 13 *Cornell LQ* 8, 43. J.S. Mill, *On Liberty* (1859) ch 5, ‘trade is a social act’.

271 TFEU Arts 9, 119–150 and TEU Art 3(3). Bank of England Act 1998 ss 1, 11–13 and 19.

272 Executive Office of the President, *Artificial Intelligence, Automation and the Economy* (December 2016) 28–44.

273 E. McGaughey, *A Casebook on Labour Law* (Oxford: Hart, 2019) ch 16.

274 J.L. Delaney, ‘The Robot That Takes Your Job Should Pay Taxes, Says Bill Gates’ (17 February 2017) Quartz.

275
*Walkovszky v Carlton*, 223 NE2d 6 (NY 1966).

276 See the Product Liability Directive 85/374/EEC, A. Bertolini, ‘Robots as Products: The Case for a Realistic Analysis of Robotic Applications and Liability Rules’ (2013) 5(2) *Law, Innovation and Technology* 214.

277 cf B. Gates, *The Road Ahead* (1996) 19 and P Piff, ‘Does Money Make You Mean?’ (2013) *TEDxMarin*.

278 E.J. Hobsbawm, *The New Century: In Conversation with Antonio Polito* (London: Abacus, 2000).

## Notes

I am very grateful to Jason Hickel, Aida Ponce, Andrea Bertolini, Simon Deakin, Adam Coutts, Christopher Markou, Liv Jores, James Hurrell, Gavin Kitching, Brendan Burchell, Shaun Hargreaves Heap, Julian Huppert, Jed Kroncke, Michael Osborne, Jeremias Prassl and an anonymous reviewer. All charts are free to copy with acknowledgement. No robots or electric sheep were harmed in writing this article.

